# Genomic evidence indicates small island-resident populations and sex-biased behaviors of Hawaiian reef Manta Rays

**DOI:** 10.1186/s12862-023-02130-0

**Published:** 2023-07-08

**Authors:** Jonathan L. Whitney, Richard R. Coleman, Mark H. Deakos

**Affiliations:** 1 National Oceanic and Atmospheric Administration, Pacific Islands Fisheries Science Center, Honolulu, Hawaiʻi USA; 2 Department of Marine Biology and Ecology, Rosenstiel School of Marine, Atmospheric, and Earth Sciences, University of Miami, Miami, FL USA; 3 Hawai’i Association for Marine Education and Research, Lahaina, Maui, Hawai’i USA

**Keywords:** Hawaiian Islands, Philopatry, Devil rays, Genetic connectivity, Genomics, Mitogenome, Dispersal

## Abstract

**Background:**

Reef manta rays (*Mobula alfredi*) are globally distributed in tropical and subtropical seas. Their life history traits (slow growth, late maturity, low reproductive output) make them vulnerable to perturbations and therefore require informed management strategies. Previous studies have reported wide-spread genetic connectivity along continental shelves suggesting high gene flow along continuous habitats spanning hundreds of kilometers. However, in the Hawaiian Islands, tagging and photo-identification evidence suggest island populations are isolated despite proximity, a hypothesis that has not yet been evaluated with genetic data.

**Results:**

This island-resident hypothesis was tested by analyzing whole mitogenome haplotypes and 2048 nuclear single nucleotide polymorphisms (SNPs) between *M. alfredi* (n = 38) on Hawaiʻi Island and Maui Nui (the 4-island complex of Maui, Molokaʻi, Lānaʻi and Kahoʻolawe). Strong divergence in the mitogenome (*Φ*_*ST*_ = 0.488) relative to nuclear genome-wide SNPs (neutral *F*_*ST*_ = 0.003; outlier *F*_*ST*_ = 0.186), and clustering of mitochondrial haplotypes among islands provides robust evidence that female reef manta rays are strongly philopatric and do not migrate between these two island groups. Combined with restricted male-mediated migration, equivalent to a single male moving between islands every 2.2 generations (~ 64 years), we provide evidence these populations are significantly demographically isolated. Estimates of contemporary effective population size (*N*_*e*_) are 104 (95% CI: 99–110) in Hawaiʻi Island and 129 (95% CI: 122–136) in Maui Nui.

**Conclusions:**

Concordant with evidence from photo identification and tagging studies, these genetic results indicate reef manta rays in Hawaiʻi have small, genetically-isolated resident island populations. We hypothesize that due to the Island Mass Effect, large islands provide sufficient resources to support resident populations, thereby making crossing deep channels separating island groups unnecessary. Small effective population size, low genetic diversity, and k-selected life history traits make these isolated populations vulnerable to region-specific anthropogenic threats, which include entanglement, boat strikes, and habitat degradation. The long-term persistence of reef manta rays in the Hawaiian Islands will require island-specific management strategies.

**Supplementary Information:**

The online version contains supplementary material available at 10.1186/s12862-023-02130-0.

## Background

Reef manta rays (*Mobula alfredi*) are planktivorous elasmobranchs that inhabit tropical and subtropical oceans between north and south latitudes of about 35 degrees [1–4]. Reef manta rays are known to have a strong affinity to specific coastal reef habitats [4–17], and spend most of their time at depths less than 50 m [11] feeding and visiting cleaning stations, while making occasional visits to nearshore pelagic habitats for foraging [6, 18]. Several discrete populations worldwide are reported to be in decline in large part due to anthropogenic threats including fishing and loss of coral reef habitat [3, 9, 19–22]. In addition, their conservative life history traits of slow growth, late maturity, and low fecundity [3, 23–26] can hinder recovery [21, 27] and contribute to their Vulnerable to Extinction status on the IUCN Red List of Threatened Species [28]. Island populations may be particularly vulnerable as remote archipelagos are more likely to be demographically and genetically isolated. However, in most island regions we have limited knowledge of genetic connectivity, effective population size, and patterns of migration, which will be critical to effective conservation and management of manta ray populations.

In the past decade, genetic studies have revealed phylogenetic and geographic partitioning both among and within species of manta rays. Phylogenetic studies have provided robust support for the species level discrimination of *M. alfredi* from oceanic manta rays (*M. birostris*) and their relationship in the family Mobulidae [29–32]. For reef manta rays, strong genetic differentiation across ocean basins suggests that large areas of open ocean are effective barriers to long distance dispersal [31, 33–35]. Until recently, the few studies that have examined population genetic structure on smaller regional scales detected no reductions in gene flow within archipelagos [33, 36] or along continuous continental coastlines spanning up to ~ 400 km [33, 34]. However, Lassauce et al. [35] provided the first evidence of fine-scale genetic differentiation between reef manta ray aggregation sites on two islands of New Caledonia. While this demonstrates the potential for restrictions in gene flow between island groups, the scale at which gene flow occurs among reef manta ray aggregation sites remains unclear for most populations.

**Fig. 1:**
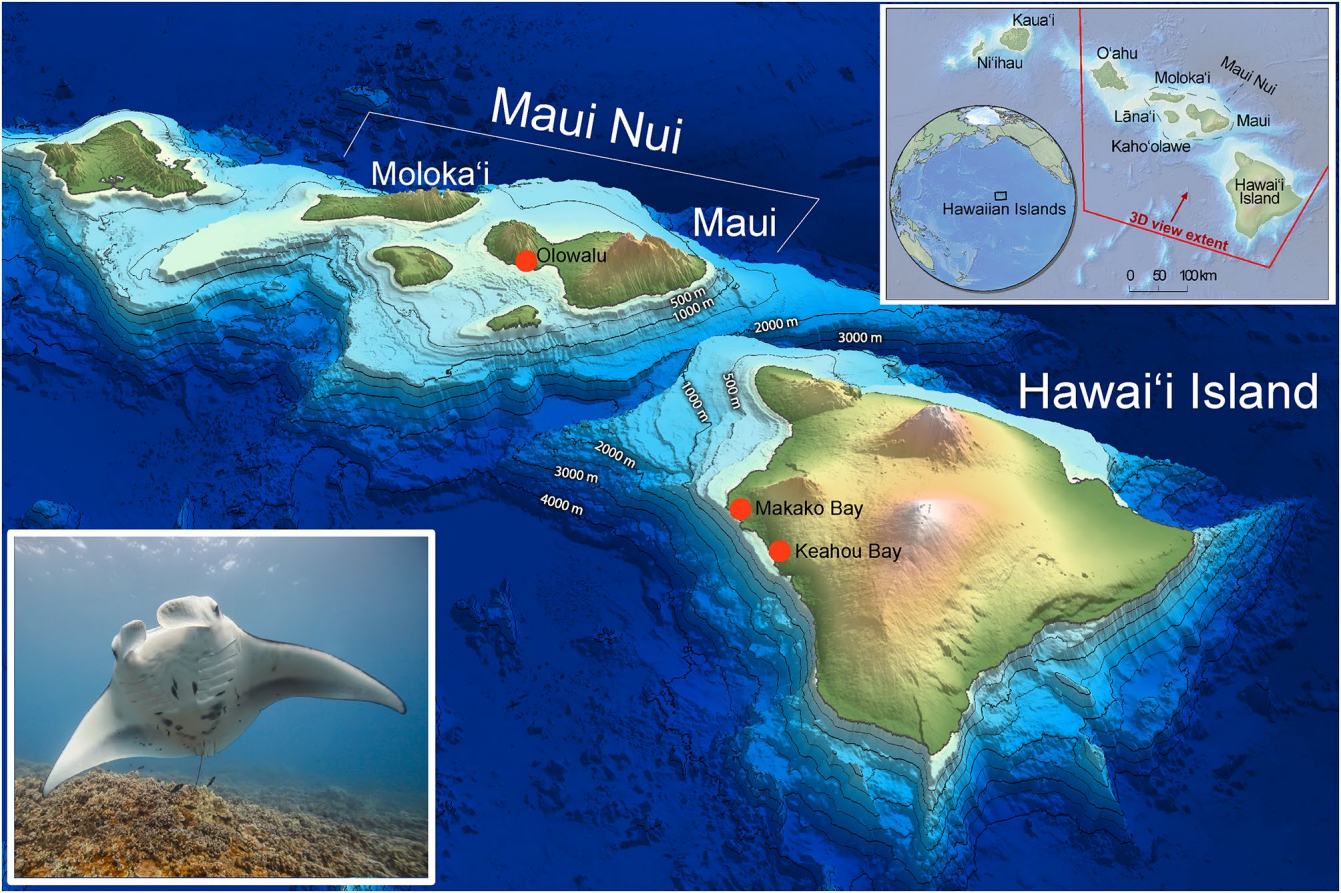
Map of collection sites in the Hawaiian Islands with inset highlighting position in the Pacific Ocean Basin and location within the Hawaiian Archipelago. Bathymetry shows contour lines at 500 m intervals. Red circles note tissue sample collection locations at aggregation sites on Maui Island and Hawaiʻi Island. Maui, Molokai, Lānaʻi and Kahoʻolawe make up the 4-island complex of Maui Nui. Map credit: Joey Lecky. Reef manta ray on Olowalu, Maui (photo credit: Mark Deakos)

In Hawaiʻi, reef manta rays occur throughout the archipelago [37], but are frequently observed and well-studied at one aggregation site on Maui Nui and two on Hawaiʻi Island (Fig. 1). Regular photo-identification cataloging of the manta ray population along the western “Kona” coast of Hawaiʻi Island (hereafter referred to as “Hawaiʻi Island”) by the Manta Pacific Research Foundation [38] has logged 318 unique individuals with photos dating back to 1979. The Maui Nui reef manta ray, which includes individuals from the islands of Maui, Molokaʻi, Lanaʻi and Kahoʻolawe, has been well studied since 2005 with some photo-identifications dating back to 1990. The Maui Nui manta ray photo-identification catalog consists of 600 unique individuals to date [39], which have demonstrated regular movements between the 4-island Maui Nui complex [9] and are therefore presumed to be a single population. No evidence exists from either tagged individuals [9, 14] or from photo-identification matches [9] that reef manta rays cross the 48 km ‘Alenuihāhā Channel that separates Maui Nui and Hawaiʻi Island, however no formal genetic evaluation has been conducted.

Here, we test the hypothesis suggested by Deakos et al. [9] that each island group supports a demographically independent population of reef manta ray. To test the island-resident hypothesis, nuclear genome-wide single nucleotide polymorphisms (SNPs) and whole mitochondrial genomes were genotyped from 38 individuals of both populations (Hawaiʻi Island = 18, Maui Nui = 20). Using this population genomics approach, we aim to determine the magnitude and direction of gene flow among populations and explore the role of sex-biased dispersal and estimates of effective population size (*N*_*e*_) to gauge population size and resilience. Overall, this study aims to provide insight into genetic stock structure and demographic parameters that can be used to inform management of reef manta rays in Hawaiʻi.

## Results

### Mitogenome assembly and diversity

Mitogenomes were successfully assembled and haplotyped in 34 individuals, with an average sequencing coverage of 82.6x (min = 16, max = 366) (Fig. S2) and high-quality base calls across 86.2% of the mitogenome (min = 62.4%, max = 99.4%)(see Additional File 4 for summaries). Across individuals, an average of 10,969 reads (range: 2,802 − 45,143) were mapped to the reference mitogenome from M03 (OP562409.1; described in [40]). The 34 individuals were haplotyped at nine variant sites (≥ 4x coverage) with an average of 88% of variable allele calls (mean calls per individual was 7.8 of 9 variable sites). Mean read depth (across individuals) per variant site was at least 33x (grand mean = 95.7x). All nine variable sites were biallelic and segregating (all transitions, no transversions) with three synonymous changes and six replacement sites. No insertions or deletions were included in the final variant dataset; however, the control region is rich in AT-repeats that were difficult to align and could contain INDELs that are not represented in this dataset.

**Fig. 2:**
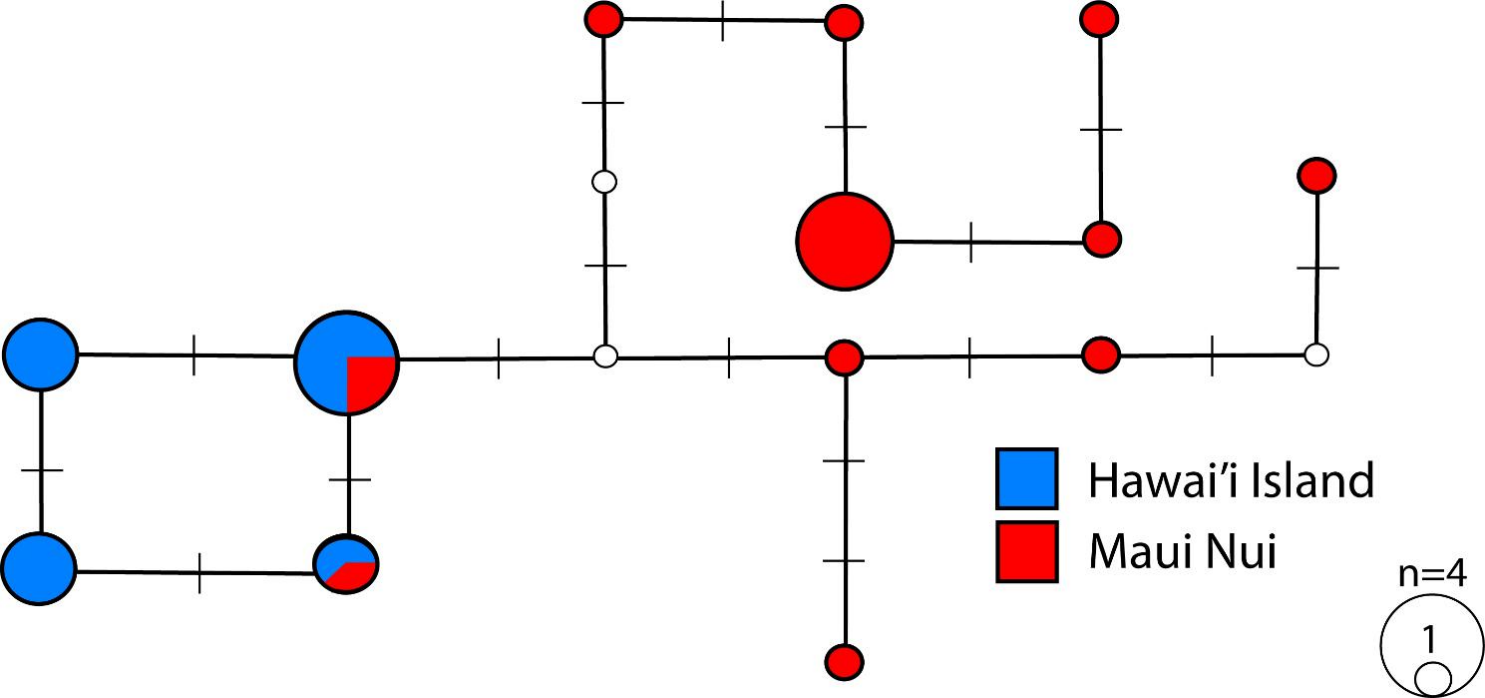
Median-joining network representing *Mobula alfredi* mitogenome haplotypes across Hawaiʻi Island (blue) and Maui Nui (red). Size of the circle represents the frequency of individuals belonging to each haplotype (1 to 4). Open circles represent unsampled haplotypes. Dashes represent the number of mutational changes between each haplotype (one dash is equal to a single base-pair change)

Mitochondrial molecular diversity indices are summarized in Table 1. Thirteen haplotypes were observed among 34 reef manta rays from the two island groups (Fig. 2). Overall nucleotide diversity was 0.00018 and haplotype diversity was 0.877. Within each population, Hawaiʻi Island had only two variable sites and Maui Nui had nine variable sites. There were no fixed differences, two shared mutations, seven unique mutations (all unique to Maui Nui), and no unique mutations to Hawaiʻi Island. Maui Nui had roughly 3-times higher genetic diversity (*π* = 0.00017), number of haplotypes (*H* = 11) and average number of nucleotide differences (*K* = 3.02) compared to Hawaiʻi Island (*π* = 0.00005, *H* = 4, *K* = 0.98). The control region was the most diverse single region with four variable sites, and the remaining five variable sites were spread among four genes: NADH5 (2), NADH4 (1), 16S ribosomal (1), and cytochrome *b* (1) (Table 2). The remainder of mitochondrial genes did not exhibit any variation across individuals.

**Table 1:** Genetic diversity indices for mitochondrial genomes of Hawaiian *Mobula alfredi* across islands and overall. Number of individuals (*N*), Number of variable sites (*S*), Number of Haplotypes (*H*), Haplotype diversity (*H*_*d*_), Nucleotide diversity (*π*), Average number of nucleotide differences (*K*), Tajima’s *D*, Fu’s *F*_*S*_, and Fu and Li’s D* (FLD*). Significance levels: NS = not significant *P* > 0.05; * = *P* < 0.05; ** *P* < 0.01.

Island	*N*	*S*	*H*	*H*_*d*_ ± SD	*π* ± SD	*K*	Tajima’s *D*	Fu’s *F*_S_	FLD*
Hawaiʻi Island	16	2	4	0.742 ± 0.073	0.00005 ± 0.00001	0.98	1.53 (NS)	-0.30 (NS)	0.91 (NS)
Maui Nui	18	9	11	0.856 ± 0.079	0.00017 ± 0.00002	3.02	0.55 (NS)	-4.44 (**)	1.35 (NS)
Overall	34	9	13	0.877 ± 0.034	0.00018 ± 0.00001	3.31	1.53 (NS)	-3.17 (NS)	1.32 (NS)

**Table 2:** Pairwise differentiation between *Mobula alfredi* populations across mitochondrial genes with variant sites and whole mitogenomes. Significant *P*-values are in bold, significance levels were adjusted using Bonferroni correction.

Mitochondrial Gene	*Φ*_*ST*_	*P*	Variable Sites	Private Alleles (Maui-Nui/Hawaii)
16S ribosomal	0.731	**0.0003**	1	1/0
NADH4	0.544	**0.0005**	1	1/0
NADH5	0.169	0.054	2	1/0
Cytochrome *b*	0.608	**< 0.0001**	1	1/0
Control Region	0.557	**< 0.0001**	4	3/0
Whole Mitogenome	0.488	**< 0.0001**	9	7/0

Neutrality tests calculated from the mitogenome were not significant (Table 1), except for Fu’s *F*_*S*_ in Maui Nui (*F*_*S*_ = -4.44, *P* = 0.009), which suggests a recent population expansion among the Maui Nui population. This result combined with the pattern that all unique variable sites were restricted to the Maui Nui population provides evidence that Hawaiʻi Island is an ancestral population, which has since expanded northward to Maui Nui. Among island populations, we found the mean number of nucleotide differences (*K*_*xy*_ = 4.44), mean number of nucleotide substitutions per site (*D*_*xy*_ = 0.00024), mean number of net substitutions per site (*D*_*a*_ = 0.00013), and nucleotide divergence (*K* = 0.00024).

### Differentiation across the mitogenome

The median-joining mitogenome haplotype network revealed thirteen haplotypes among two haplogroups coinciding with island populations (Fig. 2). Based on mtDNA haplotype frequencies, we found significant genetic structure among the two island groups (AMOVA *Φ*_*ST*_ = 0.488, *P* < 0.0001, Table 3). Genetic differentiation in the five mitochondrial regions exhibiting variation (i.e., with variable sites) among haplotypes was consistently high and significant (*Φ*_*ST*_ = 0.544–0.731, *P* < 0.001) for 4 of 5 genes including 16S, NADH4, cytochrome *b*, and the control region (Table 2). Only NADH5 showed less structure and was not significant (*Φ*_*ST*_ = 0.169, *P* = 0.054). This degree of differentiation suggests the existence of strong barriers to gene flow between Maui Nui and Hawaiʻi Island populations. Furthermore, the presence of a Hawaiʻi Island haplotype from a single individual in Maui Nui (Fig. 2) supports the direction of exportation of diversity from Hawaiʻi Island to Maui Nui.

**Table 3:** Results of analysis of molecular variance (AMOVA) for *Mobula alfredi* using whole mitogenome haplotypes. Data include % of variation, degrees of freedom (df), sum of squares (SS), and fixation statistic.

Source of Variation	Nested in	%	df	SS	*Φ*_*ST*_	*P*-value
Among Individuals	Population	51.25	32	26.52		--
Among Populations	--	48.75	1	14.19	0.488	< 0.0001

**Fig. 3:**
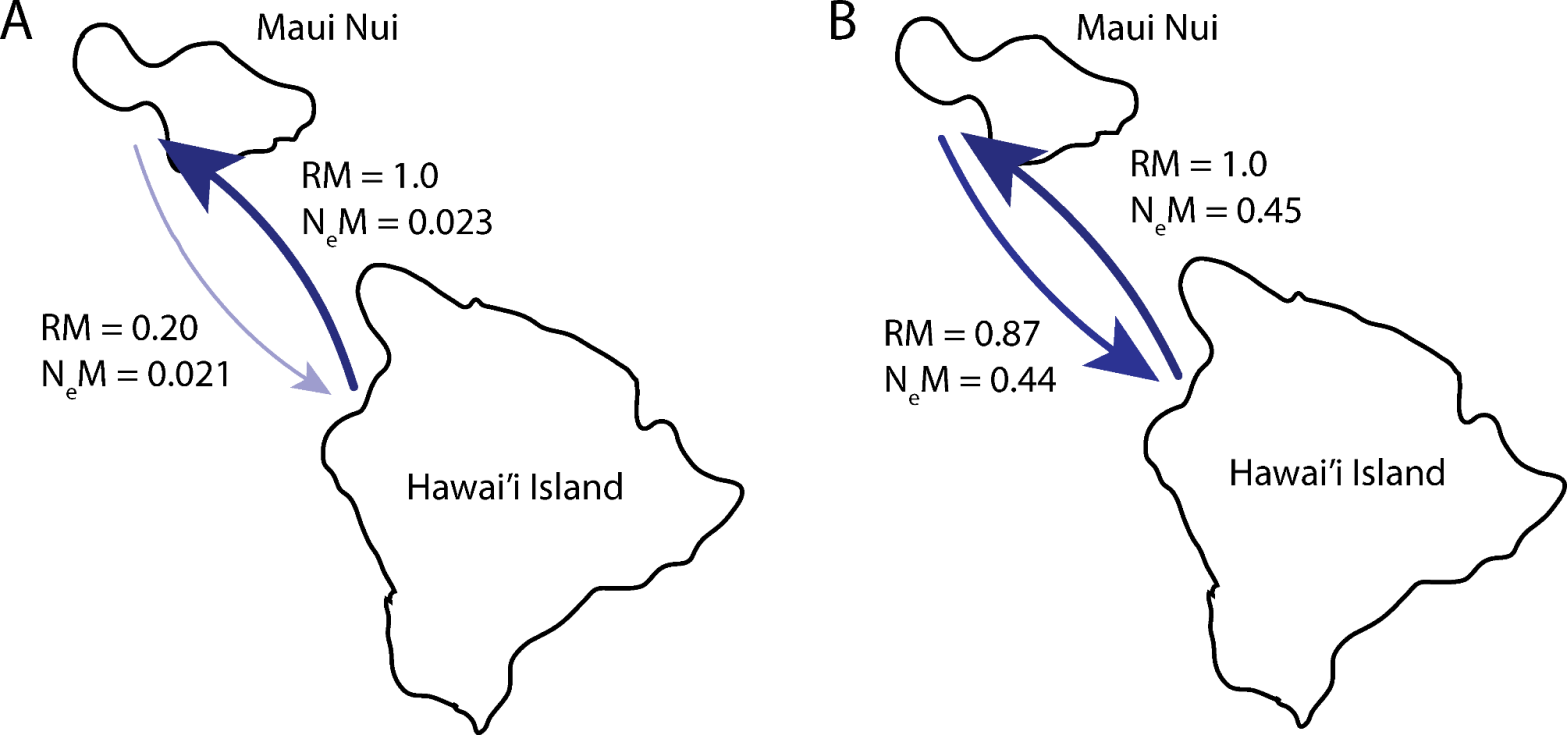
Migration network for *Mobula alfredi* populations in Maui Nui and Hawaiʻi Island using **(A)** mitogenomes and **(B)** neutral nuclear loci. Arrows indicate the direction and magnitude of migration levels, with the darker/thicker arrows showing stronger rates of migration relative to thinner/lighter arrows. RM, relative migration; *N*_*e*_*M*, effective number of migrants per generation estimated from MIGRATE-N

Coalescence-based migration estimates determined by MIGRATE-N showed an overall pattern of net migration from Hawaiʻi Island to Maui Nui (Fig. 3). The mitogenome showed an effective number of migrants per generation (*N*_*e*_*M*) of 0.023 (95% CI: 0.011–0.043) and 0.021 (95% CI: 0.008–0.047) moving northwesterly from Hawaiʻi Island to Maui Nui and southeasterly from Maui Nui to Hawaiʻi Island, respectively (Table 4). Migration estimates overall gene flow (mean *N*_*e*_*M* = 0.022; 95% CI: 0.01–0.045) to be equivalent to 1 female migrant moving between these island groups every 45 generations, or approximately 1305 years, based on the estimated 29-year generation time [10, 25, 28]. For every one migrant from Hawaiʻi Island to Maui Nui, the relative migration network analysis estimated that there were 0.20 migrants from Maui Nui to Hawaiʻi Island (Fig. 3), further supporting net migration from Hawaiʻi Island northwesterly to Maui Nui.

**Table 4:** Migration estimates (*N*_*e*_*M*, effective number of migrants per generation) derived from neutral nuclear loci and mitogenome sequence data. Numbers in parentheses represent 95% confidence intervals.

	*N*_*e*_*M* (effective number of migrants per generation)		
Dataset	Hawaiʻi Island to Maui Nui	Maui Nui to Hawaiʻi Island	Overall
Nuclear	0.45 (0.06–0.88)	0.44 (0.18–0.87)	0.45 (0.12–0.88)
Mitogenome	0.023 (0.011–0.043)	0.021 (0.008–0.047)	0.022 (0.010–0.045)

### Nuclear genome scans

A total of 2048 filtered and informative nuclear genome-wide SNPs were successfully genotyped in 38 individuals. Employing the OutFLANK approach across island groups, we detected 10 SNPs putatively under divergent selection (Fig. S3) and 2038 neutral nuclear SNPs. For all subsequent analyses using nuclear loci, we analyzed the neutral loci (2038 SNPs), and outlier loci (10 SNPs) separately. While we also conducted all analyses using these two datasets combined (i.e., all 2048 nuclear loci) we do not present those results in the main text as they were very similar to neutral loci datasets and presented no change in inference. Nuclear molecular diversity indices are summarized in Table 5. For neutral nuclear loci, the mean number of alleles per locus, effective number of alleles, and the observed heterozygosity were all higher in Maui Nui (*N*_a_ = 2.022, *N*_eff_ = 1.594; *H*_O_ = 0.508) compared to Hawaiʻi Island (*N*_a_ = 2.018, *N*_eff_ = 1.555; *H*_O_ = 0.471). The same pattern was true and more pronounced for the outlier loci with Maui Nui (*N*_a_ = 1.800, *N*_eff_ = 1.508; *H*_O_ = 0.446) presenting higher diversity indices than Hawaiʻi Island (*N*_a_ = 1.700, *N*_eff_ = 1.256; *H*_O_ = 0.221). Inbreeding coefficients revealed that the influence of inbreeding is negligible across all populations for both neutral and oultier loci datasets (Table 5).

**Table 5:** Molecular diversity indices for populations of *Mobula alfredi* based on two nuclear datasets: neutral loci (2038 SNPs) and outlier loci (10 SNPs). Number of individuals genotyped (*n*), average number of alleles per locus (*N*_a_), effective number of alleles (*N*_eff_), Observed heterozygosity (*H*_O_), heterozygosity between populations (*H*_S_), total heterozygosity (*H*_T_), and inbreeding coefficient (*G*_IS_) are presented.

Dataset	Sample Location	*n*	*N*_a_	*N*_eff_	*H*_O_	*H*_S_	*H*_T_	*G*_IS_
Neutral loci	Hawai’i Island	18	2.018	1.555	0.471	0.343	--	-0.372
	Maui Nui	20	2.022	1.594	0.508	0.358	--	-0.422
	All populations	38	2.023	1.568	0.490	0.350	0.351	-0.397
Outlier loci	Hawai’i Island	18	1.700	1.256	0.221	0.177	--	-0.247
	Maui Nui	20	1.800	1.508	0.446	0.299	--	-0.494
	All populations	38	2.000	1.321	0.334	0.238	0.269	-0.402

**Fig. 4:**
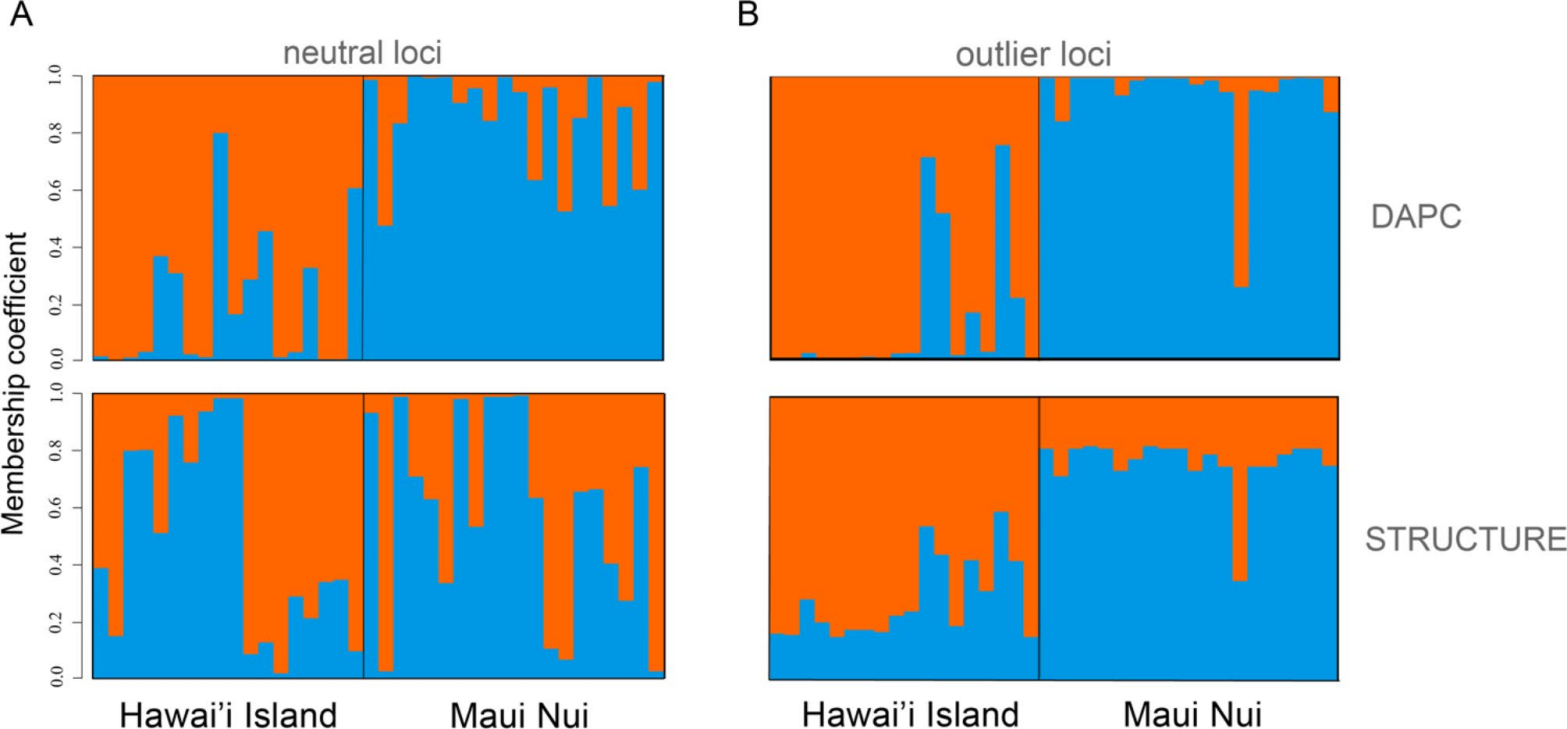
Assignment tests for Hawaiian populations of *Mobula alfredi* using nuclear loci: **(A)** neutral SNPs (2038) and **(B)** outlier SNPs (10) from both DAPC (top) and STRUCTURE (bottom) analyses. Each bar represents one individual genotype, colors correspond to the inferred evolutionary cluster to which they were assigned when K = 2. Red is representative of the probability of assignment with the Hawai’i Island population and blue is representative of the probability of assignment with the Maui Nui population

Population structure between Maui Nui and Hawaiʻi Island was detected in both neutral and outlier nuclear loci (Table 6; Fig. 4). Genetic differentiation of the neutral nuclear loci (*F*_ST_ = 0.003, *P* = 0.033) was significant but notably weaker relative to the mitochondrial genome (*Φ*_*ST*_ = 0.488). The STRUCTURE analysis based on outlier loci recovered segregating populations between island groups (K = 2, Table S5), which coincided well with the DAPC analyses (Fig. 4). The STRUCTURE analysis based on neutral loci also resolved two populations (K = 2, Fig. S4, Table S5), but the overall pattern showed a high degree of admixture and the clustering signal between islands was much lower than was observed in the oulier loci (Fig. 4). The DAPC analyses detected a stronger clustering pattern among island groups compared with STRUCTURE, but overall results coincide well from both tests. The AMOVA and STRUCTURE output using all 2048 nuclear loci (combining neutral and outlier) produced similar patterns to the neutral loci (Table S6; STRUCTURE results not included here). In contrast to the relatively low genetic differentiation at neutral loci (*F*_ST_ = 0.003), differentiation at outlier loci was more than 60x higher (*F*_ST_ = 0.186, *P* < 0.001), and showed a clear pattern of clustering among islands, yet with still some degree of admixture (Fig. 4).

**Table 6:** Results of the analysis of molecular variance (AMOVA) of neutral and outlier nuclear loci in Hawaiian populations of *Mobula alfredi*. Bolded values denote significance at *P* < 0.05.

Dataset	Source of Variation	F-statistic	% Variation	F-value	Std. Dev.	P-value
Neutral loci	Within Individual	*F*_*IT*_	1.366	-0.366	0.005	--
	Among Individual	*F*_*IS*_	-0.369	-0.37	0.005	1.000
	Among Population	*F*_*ST*_	**0.003**	**0.003**	**0.001**	**0.033**
Outlier loci	Within Individual	*F*_*IT*_	1.131	-0.131	0.047	--
	Among Individual	*F*_*IS*_	-0.316	-0.388	0.061	1.000
	Among Population	*F*_*ST*_	**0.186**	**0.186**	**0.012**	**0.001**

We attempted to annotate RAD contigs containing outlier loci to explore potential ecologically relevant functions. Of 10 contigs containing outlier SNPs identified with OutFLANK, three blasted without hits (i.e., no sequence counterpart in GenBank), three had blast hits to known sequences but were unmapped, and the remaining four were mapped and annotated with gene ontology terms (Table S2). Sequence similarity scores to annotated genes were relatively low (< 75%), and there were no obvious patterns of enrichment or ecologically relevant adaptive functions of the four genes associated with outlier loci.

Coalescence-based migration estimates determined by MIGRATE-N varied among datasets but showed an overall pattern of net migration from Hawaiʻi Island to Maui Nui (Table 4; Fig. 3). Migration estimates from neutral loci showed relatively low migration rates, that are only slightly higher in the northwesterly direction *N*_*e*_*M* = 0.45 (95% CI: 0.06–0.88) compared to *N*_*e*_*M* = 0.44 (95% CI: 0.18–0.87) in the southeasterly direction. The magnitude of migration varied across nuclear datasets, however the overall pattern of net migration from Hawaiʻi Island to Maui Nui was concordant across all datasets (Table 4). Based on nuclear loci, for every one migrant from Hawaiʻi Island to Maui Nui, the relative migration network analysis estimated that there were 0.87 migrants from Maui Nui to Hawaiʻi Island (Fig. 3). Migration analysis on neutral nuclear loci estimates overall gene flow (mean *N*_*e*_*M* = 0.45; 95% CI: 0.12–0.88) equivalent to 1 effective migrant moving between these island groups every 2.2 generations, or approximately every 64 years, based on the estimated 29-year generation time. Migration estimates were 20 times greater for nuclear loci than the mitogenome. The patterns of net migration in both the nuclear and mitogenome, coupled with the results of the haplotype network, which revealed the presence of Hawaiʻi Island haplotypes in Maui Nui (but not the inverse), supports a Hawaiʻi Island to Maui Nui net direction of migration.

Estimates of contemporary genetic effective population size (*N*_*e*_) based on neutral nuclear loci resulted in *N*_*e*(raw)_ estimates of 104 (95% CI: 99–110) for Hawaiʻi Island and *N*_*e*(raw)_ = 129 (95% CI: 122–136) for Maui Nui populations. *N*_*e*(adj)_ estimates were found to be slightly less conservative with Hawaiʻi Island and Maui Nui, estimated to be 122 (95% CI: 110–127) and 155 (95% CI: 141–157), respectively.

## Discussion

We investigated the fine-scale genetic structure of reef manta rays (*Mobula alfredi*) in the Hawaiian Islands using a combination of genome-wide nuclear loci and whole mitogenome sequences. Our assessment of genetic connectivity revealed that patterns of gene flow are associated with the geographic separation of island groups, with significant differentiation observed in the mitochondrial genome (*Φ*_*ST*_ = 0.488, *P* < 0.0001) as well as neutral (*F*_*ST*_ = 0.003, *P* = 0.033) and outlier loci (*F*_*ST*_ = 0.186, *P* = 0.001) across the nuclear genome (Figs. 2, 4 and 3). This pattern aligns with the expectations for island-associated populations of reef manta rays, which are known to have limited home ranges and high site fidelity [6, 7, 11, 13, 41]. Our results of fine-scale genetic structure among island groups in Hawai’i are consistent with that of Lassauce et al. [35] from New Caledonia and reinforce evidence that insular populations can be genetically isolated at small spatial scales (< 100 km). Furthermore, we describe new insights regarding patterns of sex-biased migration and genetic connectivity and discuss potential mechanisms that may be limiting the dispersal of reef manta rays between islands.

### Patterns of dispersal and connectivity

#### Sex-biased dispersal among islands

Patterns of genetic differentiation of mitochondrial genomes and nuclear outlier loci both show a clear clustering among island populations (Figs. 2 and 4B). Outlier loci can reflect genomic regions associated with local adaptive differences [42, 43]. Strong differentiation at these regions putatively under selection indicates that localized selection could be overpowering the homogenizing force of male dispersal. These patterns of male-mediated dispersal and signatures of local selection are consistent with the hypothesis advanced by Portnoy et al. [44] that the combination of philopatric females and dispersing males may favor local adaptation by simultaneously allowing dispersal and the localized sorting of adaptive alleles.

Strong divergence in the maternally-inherited mitogenome (*Φ*_ST_ = 0.488) relative to the biparentally-inherited nuclear genome (*F*_ST_ = 0.003) is robust evidence that female reef manta rays are strongly reproductively philopatric. This signal of differentiation and geographic clustering of mitochondrial haplotypes (Fig. 2) indicates the existence of strong barriers to gene flow between Maui Nui and Hawaiʻi Island, with female reef manta rays remaining resident to, and reproducing predominantly around, the island where they were born. Comparatively, the low, but significant divergence in neutral loci across the nuclear genome indicates reduced gene flow mediated by weak male-biased dispersal between island groups. Coalescence-based migration analysis estimates movement between islands equivalent to 1 individual migrant every 64 years for males (nuclear loci) and 1305 years for females (mitogenome) (Table 4). The combination of restricted female- and male-mediated migration provides evidence these populations are demographically isolated. This asymmetry in spatial genetic patterns between nuclear loci and mitochondrial haplotypes among reef manta rays is consistent with female philopatry and weak male-biased dispersal [45, 46], which has been documented in batoids [47–50] and sharks [51–54]. Broadly, this molecular evidence for male-biased dispersal in reef manta rays adds further support to sex-biased dispersal as a recurrent pattern in viviparous elasmobranchs (reviewed in Phillips et al. [55]).

Female reproductive philopatry is common among elasmobranchs and widespread across batoids including several species of stingrays [56], skates [56, 57], and sawfish [47, 50, 55]. Previous studies (reviewed in Flowers et al. [58]) have provided compelling evidence of site fidelity and residency in *M. alfredi* that are consistent with philopatry [6, 8–10, 12, 15, 59–62]. However, this study is the first to confirm reproductive philopatry in reef manta rays using genetic evidence.

#### Evidence supporting the island-resident hypothesis

Here, we review evidence evaluating hypotheses for the drivers of population breaks between island groups. We provide support for the hypothesis suggested by Deakos et al. [9], that large islands provide sufficient coastal resources to support resident populations of reef manta rays, thereby making inter-island dispersal unnecessary.

The genetic patterns presented here are in concordance with evidence from photo-identification and tagging studies and thus confirm that Maui Nui and Hawaiʻi Island reef manta ray populations are distinct population stocks with restricted movement between them. First, no photo-identification matches have been made between catalogs on Hawaiʻi Island, which to date contains 318 unique individuals (1979–2023; [38]), and within Maui Nui, which contains 600 unique individuals (2005–2023; [39]). The number of individuals in the catalog that have perished since their last sighting is unknown. Second, active tracking and satellite tagging of 53 unique individuals (40 in Clark [14], 13 in Deakos et al. [9] and Deakos, *unpublished*) demonstrated that reef manta rays readily moved between the islands of Maui Nui but did not migrate to Hawaiʻi Island (and vice-versa). Therefore, photo-identification and tagging studies all provide no evidence of animal movements between Maui Nui and Hawaiʻi Island [9, 14].

Availability of resources may be driving this limited movement of reef manta rays between islands. Reef manta rays forage for zooplankton in generally nutrient-poor oligotrophic waters in tropical and subtropical oceans. To meet energetic needs of this large planktivore, reef manta rays require high densities of planktonic prey [63]. Islands provide localized hotspots of productivity compared to surrounding pelagic waters. This fertilizing effect of islands, known as the Island Mass Effect [64], is driven by several mechanisms including island-induced mixing, nutrient flux from freshwater runoff, mesoscale eddies, and increased internal wave activity, all of which combine to enhance phytoplankton productivity near islands [65]. Biological production scales with total reef area, thus larger islands with greater reef area exhibit increased productivity enhancements that translate up the food web [65].

At small scales, reef manta ray feeding events coincide with localized high biomass of zooplankton driven by fine-scale oceanographic processes, such as strong tidal currents interacting with island topography [5, 63], cold-water bores created by breaking internal waves [13], and surface slicks generated by Langmuir cells [66]. On Hawaiʻi Island, surface slicks are ubiquitous and prevalent along the western coastline and have been found to accumulate dense concentrations of zooplankton [67]. These features, which are driven by a variety of mechanisms including internal waves and Langmuir cells [68], have been correlated with reef manta ray feeding events in other regions [66] and could provide enhanced foraging opportunities near islands.

Collectively, these fine-scale and meso-scale oceanographic features, all enhanced by the Island Mass Effect, provide more biomass and greater predictability of planktonic prey near large islands [65]. Reliable coastal resources likely eliminate the need to travel to other islands to forage and thus could be driving the strong population differentiation among islands for insular reef manta rays.

Within the Hawaiian Archipelago, similar patterns of population and genetic breaks between Hawaiʻi Island and Maui Nui have been observed in several wide-ranging insular species including common bottlenose dolphins [69, 70], spinner dolphins [71], rough-toothed dolphins [72, 73], and pantropical spotted dolphins [74]. These studies similarly cite the increased productivity around the Hawaiian Islands due to the Island Mass Effect as the leading explanation driving patterns of high site fidelity and population differentiation among neighboring islands [69, 70, 72, 73].

We reject the alternative hypothesis that the lack of exchange between island groups results from isolation by distance. The shortest distance between Maui Nui and Hawaiʻi Island is only 49 km, and linear movements up to 91 km have been documented elsewhere in Hawaiʻi [14] and more than 200 km between nearby islands in other archipelagos (Table S3) [11, 12, 16, 60, 75, 76]. Along continental shelves with continuous coastlines, linear movements over 500 km are common [18, 60, 77, 78].

There is evidence that deep-channel crossings create habitat breaks that can be barriers to dispersal for reef manta rays, particularly when separating large islands. First, the deepest area transited over following an acoustically tagged reef manta ray in Hawaiʻi was 360 m in Maui Nui [9] and 300 m off Hawaiʻi Island [14]. Tracked individuals generally followed the bottom contour depth, remaining in relatively shallow water with maximum recorded dive depths of 218 m [14] and 308 m (Deakos, *unpublished*). Hawaiʻi Island and the Maui Nui island complex are large, high volcanic islands separated by the ʻAlenuihāhā channel, which has a minimum crossing depth of 1900 m, and plummets to depths > 4700 m on both eastern and western sides (Fig. 1). Second, the few inter-island movements over deep-water that have been recorded globally all occur between small atolls with island areas less than 46 km^2^ (Table S3) [11, 12, 41, 75]. For example, in French Polynesia, Carpentier et al. [41] reported crossings between Bora Bora and Maupiti, a channel similar in span (50 km) and depth (> 3000 m) to the ʻAlenuihāhā Channel. However, these islands are only 0.2% of the area of Hawaiʻi Island (Table S3). Third, in contrast, regular inter-island movements up to 450 km have been recorded between relatively large islands in Indonesia that are connected by shallow shelves typically < 300 m [15, 16, 60, 62, 79]. These patterns reinforce the hypothesis that shallow channels and shelves create more continuous habitat that is crossed regularly, regardless of island size, but deep channels (> 300 m) are crossed infrequently and only when separating small islands or atolls. Altogether this suggests that the interaction of island size (as a proxy for coastline generated resources), channel depth, and philopatric behavior together play an important role in determining movement patterns and ultimately gene flow in reef manta ray insular populations. It remains unclear why deep-water inhibits movement, but could be due to increased exposure to predators, less foraging opportunities, and reduced ability to navigate via bathymetry.

#### Oceanic and archipelagic patterns of genetic connectivity

At the ocean basin scale, evidence of genetic structure among populations of reef manta rays demonstrates little potential for long-distance dispersal and migration from distant populations [33, 80]. Ocean basins are common barriers to dispersal in other elasmobranchs [81], particularly reef-associated sharks [54, 82, 83]. With that said, an opportunistic sighting of a pregnant reef manta ray at Cocos Island in the Eastern Tropical Pacific [84], nearly 6000 km from the nearest aggregation site in the Marquesas (and ~ 7500 km to Hawai’i), reminds us of their potential for oceanic dispersal, even if extremely rare.

Perhaps not surprisingly, the pattern of regional genetic structure observed in reef manta rays is in direct contrast to genetic patterns in the oceanic manta ray (*Mobula birostris*), which shows relative panmixia across their circumtropical distribution [33] albeit with indications of structure in the Eastern Tropical Pacific [85]. The contrast in reef manta rays with high site fidelity versus oceanic manta rays with wide-ranging behavior, can explain these observed differences in genetic connectivity.

Our results of genetic structure among neighboring islands within the Hawaiian Islands provides evidence of finer-scale restrictions in gene flow than observed in surveys on continental coastlines [34] and within the Maldives archipelago [33, 36]. In Mozambique, Venables et al. [34] report high genetic connectivity along ~ 400 km of continuous coastline. Similarly, Hosegood [33] detected no genetic sub-structuring among islands in the Maldives spanning up to 350 km across the archipelago. In both regions, animals move more regularly between aggregation sites [59, 80, 86], which likely explains higher gene flow than that observed among island groups in Hawaiʻi (current study) and New Caledonia [35] where inter-island movement is restricted. Our results are comparable to the fine-scale structure observed between Grande Terre (New Caledonia) and Ouvea, which are approximately ~ 120 km apart across a deep-channel (> 2000 m) [35]. Patterns from genetics and photo-identification in both regions [9, 35] indicate high site fidelity and few connections between aggregation sites, suggesting movement and gene flow across islands are restricted.

### Patterns and implications of small effective population size estimates

Our estimates of contemporary effective population size (*N*_*e*_) are 104 for Hawaiʻi Island and 129 for Maui Nui. When sampled from a mixed-age group and overlapping generations, as in this study, *N*_*e*_ is an approximate estimate of the harmonic mean of the number of breeders (*N*_*b*_) in the population over several generations [87, 88]. There are not yet robust abundance estimates of reef manta ray populations in the Hawaiian Islands (see [9, 89]), however minimum population size can be approximated using the photo-identification catalog sizes of 318 and 600 unique individuals for Hawaiʻi Island and Maui Nui, respectively. These minimum population sizes do not consider individuals that have died. A decade or more can sometimes pass between sightings of certain individuals making it difficult to determine when an individual is no longer part of the population. While no constant relationships exist between *N*_*e*_ and the minimum estimates for census size (*N*_*c*_*)* across taxa [90, 91], our results are intermediate to the only two reef manta ray *N*_*e*_ estimates published to date [34, 92]. The Yaeyaema, Japan population has an *N*_*e*_ estimated at 89 and a catalog size of 305 unique individuals [92]. The population along the southern Mozambique coast has an *N*_*e*_ estimated at 375 [34] and a catalog size of 1209 [80, 93]. Using catalog size as minimum estimates for census size (*N*_*c*_), the *N*_*e*_/*N*_*c*_ ratios in these four populations are similarly between ~ 0.2–0.3 (Table S4), suggesting that this ratio is relatively consistent among reef manta ray populations and could be useful for estimating one metric in the absence of the other.

The relatively high *N*_*e*_/*N*_*c*_ ratios observed in reef manta rays are consistent with species with Type I survivorship curves, which well characterize manta rays and other viviparous elasmobranchs [91]. Several studies have demonstrated the variation in *N*_*e*_/*N*_*c*_ among taxa is driven primarily by age-at-maturity, adult lifespan, and variation in reproductive success [94–96]. Reef manta rays have a delayed age-at-maturity (8–17 years), long lifespans up to 45 years [10, 25], and low and variable reproductive output of a single pup every 1 to 7 years for mature females [23–25, 79, 92].

We acknowledge that the relatively small sample sizes used in this study may reduce precision in our estimates of contemporary *N*_*e*_. Precision and accuracy of *N*_*e*_ estimates derived from low numbers of samples (and loci) can decline resulting in infinite parameters or upper confidence limits [97]. However, our study utilized high numbers of loci (> 2000 SNPs), which improve precision in *N*_*e*_ for small population sizes [98]. Furthermore, the *N*_*e*_ confidence limits we report are finite and relatively narrow for both Hawai’i Island (104; 95% CI: 99–110) and Maui Nui (129; 95% CI: 122–136) populations, suggesting these data have sufficient power and precision for estimating *N*_*e*_. Additionally, our relative number of samples to *N*_*e*_ estimates (13–15% of *N*_*e*_) are above the 10% of *N*_*e*_ guideline proposed by Palstra & Ruzzante [99] and similar to estimates of other elasmobranchs [100, 101]. Efforts to increase sample size and geographic coverage are ongoing and are expected to improve precision in future genetic surveys of reef manta rays in the Hawaiian Islands.

Most regions support relatively small populations of reef manta rays, typically less than 1,000 individuals. Long-term photo-identification studies have produced minimum estimates (i.e., catalog sizes) as low as 54 animals in Yap, Micronesia [102] and up to 4,411 individuals in the Maldives [86]. Larger population sizes (over 600) tend to be associated with continental shelves such as the East Coast of Africa [8], Australia [10], as well as archipelagos consisting of many islands connected by shallow water like the Maldives [59, 86], and Indonesia [16, 76]. Smaller populations tend to be associated with remote archipelagos with islands separated by deep-water including Hawai’i [9, 14], French Polynesia [41], New Caledonia [35], and Seychelles [11, 103] and generally have small home ranges. These smaller home ranges can likely be attributed to having access to sufficient coastal resources, cleaning stations, mates, and protection from predation [5, 104].

### Patterns and consequences of low genetic diversity

We conclude that the low genetic diversity observed in reef manta rays is due to low mutation rates combined with inherently small, localized populations. The low levels of genome-wide diversity observed in Hawaiian reef manta rays (Tables 1 and 5) are generally consistent across elasmobranchs (e.g., [105, 106]). For example, a comparison of whole mitogenomic diversity of the endangered speartooth shark (*Glyphis glyphis*) revealed strikingly similar patterns of genetic variation (i.e., *π =* 0.00019, *h =* 0.76; [107]) to those of *M. alfredi* (*π =* 0.00018, *h =* 0.88; Table 1). In *G. glyphis*, the low genetic diversity was attributed to low mutation rates and a low effective population size, which follow patterns in other elasmobranchs [106, 108]. Although empirical estimates of mutation rates do not yet exist for reef manta rays, the rates of mitochondrial mutations in elasmobranchs are slow relative to other taxa [109]. With concordant patterns within a variety of evolutionary distinct elasmobranchs, it is reasonable to conclude low mutation rates are naturally occurring phenomena in mobulid rays and, along with small effective population size, contributes to the low diversity observed in reef manta rays.

The concern regarding the consequences for populations with low genetic diversity is compounded when population sizes are small, as in reef manta rays. When low genetic diversity is observed in a population, it is often interpreted as an indication of inbreeding depression [94] and is thought to compromise reproductive fitness and capacity for population growth [110]. However, recent research has challenged the assumption that high levels of genetic diversity are necessary for increased fitness and survival and reframed the importance of genetic diversity when considering variation that is relevant to future environmental and climatic changes [111]. Based on patterns in other elasmobranchs and other populations of reef manta rays, low population size may be the natural state of this species and low genetic diversity may not be a suitable barometer for evaluating population health and extinction risk. There is evidence of a population expansion for the Maui Nui population based on a single metric (Table 1; Fu’s *F*_*s*_ = -4.44. *P* < 0.01), however, other neutrality tests (i.e., Tajima’s *D* or Fu and Li’s *D*) did not show support of a bottleneck or recent expansion, suggesting these Hawaiian populations have not fluctuated dramatically in size. Thus, small population sizes and low genetic diversity may be the natural biological state for this species at least in this region.

### Management implications of island-resident populations

Combined with evidence from photo-identification and tagging studies, these genetic results indicate reef manta rays in the Hawaiian Islands have small resident island populations that are significantly genetically isolated and should be managed as discrete stocks. The lack of female migration among islands means extremely little potential for replacement females to enter and establish from other islands. The relatively low levels of male-mediated migration still indicate that replacement males cannot be counted on to replenish island populations should they decline. On the ocean basin scale, evidence of genetic differentiation between Hawai’i and the South Pacific and Indian Oceans [33] provides further evidence that Hawaiian reef manta rays are demographically isolated from other regions. The high degree of residency, low genetic connectivity, and geographic isolation of the Hawaiian archipelago all suggest there is little potential for replenishment from distant aggregation sites. Together with small population size, restricted gene flow, low genetic diversity, and conservative life history traits, this leaves reef manta rays at extremely high risk to human-induced perturbations. Reef manta rays have low intrinsic growth rates due to their delayed age-at-maturity (8–17 years for females) and low fecundity [9, 28, 89]. These extremely conservative life history traits are expected to severely restrict the potential for recovery from any potential population reductions in the future.

Globally, several reef manta ray populations have been reported to be in decline [28] and has resulted in their listing on Appendix II of the Convention for the International Trade in Endangered Species of Wild Fauna and Flora (CITES) in 2013. Much of the decline has been attributed to direct manta ray hunts to provide for the global demand of gill plates [4, 8, 21, 22]. In Hawaiʻi, manta rays are neither fished intentionally nor known to be caught as bycatch in any fishery. In 2009 a state bill was passed that protects manta rays from being killed or captured. Despite these much-needed protections, sighting rates at a reliable cleaning station on the island of Maui have declined by over 90% in the past decade (Deakos, *unpublished*). Whether this is due to a reduction in population size or manta rays relocating elsewhere due to degradation of reef habitat remains unknown and requires further investigation. In addition, more than 10% of the Maui Nui population has evidence of entanglement in fishing line, primarily from shore-casting fishing gear used to target giant trevally (*Caranx ignobilis*) [9]. Injury to the cephalic fins from fishing line could negatively impact feeding efficiency and mate attraction and thus may have long-term consequences even if animals survive.

Monitoring long-term population trends and breeding stock will be important for evaluating the population level impacts of local anthropogenic threats, which include fishing line entanglement [9, 20], pollutants and contaminants [112], plastic ingestion [113], boat strikes [20, 41], pressures from commercial manta ray dive tours [14, 22], habitat degradation as a result of coastal development [86, 114], as well as projected declines in zooplankton [115] and climate change.

Further research to assist with effective management strategies should include the following. First, identifying essential habitat areas that are used for cleaning, feeding, mating, and pupping [62] could be achieved by expanding telemetry studies to develop core use density maps [7]. Second, with critical habitats defined, a focus should be made on quantifying and reducing regional anthropogenic threats, especially habitat degradation around nursery and aggregation sites, both important for reproduction. In addition, an assessment of the impact of commercial tourism activities should be conducted, including evaluating mitigations such as establishing codes of conduct [116], setting carrying capacity for boats/divers and implementing rest-periods in heavy use areas. Third, robust mark-recapture population models should be built by expanding photo-ID and acoustic tagging efforts to improve survival and abundance estimates (e.g., [117]) or adopting a modification of the standard POPAN model that incorporates per capita recruitment and transience parameters to estimate annual population sizes [76]. Fourth, genetic sampling should be expanded across the archipelago and years to evaluate gene flow, monitor changes in contemporary *N*_*e*_ [154], and assess population trends. Fifth, building population projection models that incorporate target prey availability (informed by empirical study of prey density thresholds) and examine extirpation risk can be used to predict population trends under different management and climate change scenarios. Collectively, these efforts coupled with engagement with the public and stakeholders will be critical to ensuring the long-term persistence of healthy populations of island-resident reef manta rays in the Hawaiian Islands.

Long-term stability of aggregation sites is beneficial for the stability of social structure, particularly mating behavior. In addition to benefits of cleaning, coral reef cleaning stations also serve as hubs for reproductive activity [9, 23, 25]. Degradation of nearshore coral reef habitat serving as mating aggregation sites, like in Maui [9], is expected to negatively influence reproductive success and could have long-term demographic consequences. The loss or degradation of embayments that can serve as pupping/nursery grounds [62, 113] can shift patterns of natural selection and negatively influence demography [118]. Degradation of coastal coral reefs and embayments from coastal development and climate change represents a serious threat to reef manta rays in Hawaiʻi and elsewhere.

## Conclusions

We provide evidence of strong female reproductive philopatry and weak male-mediated dispersal that indicate genetic isolation of small resident island populations of reef manta rays in Hawaiʻi. Despite the proximity of these island-associating populations, they represent two genetically distinct stocks with varying geographic characteristics. The Hawaiʻi Island population demonstrates a much tighter home range but has immediate access to deep-waters whereas the Maui Nui population shares a relatively shallow bathymetry between the 4-island Maui Nui complex. The threats affecting Maui Nui’s population may be more dependent on entanglement in coastal fishing line and habitat degradation, whereas the Hawaiʻi Island population face a different set of challenges from direct interaction with boats and divers. These distinct differences between neighboring island populations, combined with their reproductive isolation and vulnerable life history characteristics, highlight the importance of local, island-specific management strategies.

## Methods

### Sample collection

Most data were collected opportunistically while freediving or with open-circuit SCUBA, either from a boat or from a shoreline entry at known manta ray aggregation areas (Table 7; Fig. 1). Biopsy samples were collected from March 2012 to October 2015. For each manta ray encountered, attempts were made to get a photo-identification of the ventral side, a gender and age-class determination (juvenile or adult) based on clasper development in males [9, 23, 119] or mating scars and visible pregnancy in females [23], and body size measurements using paired-laser photogrammetry as described in Deakos [120]. Biopsy samples were obtained using a modified Hawaiian sling containing a stainless-steel cylindrical biopsy tip (13 mm in length x 5 mm in diameter) that extracts a sample of skin and muscle. Biopsies were taken from the caudal end of the manta ray’s disc, to avoid sampling close to the main body trunk. Samples were preserved in either 20% salt-saturated dimethyl sulphoxide (DMSO) or 95% ethanol and stored at -20 °C. Biopsy tips were washed and sterilized in a 10% bleach solution for 5 min, rinsed with fresh water, soaked for 5 min in 95% ethanol, air dried, and placed individually into small bags for reuse.

**Table 7:** Metadata for reef manta ray genetic samples including Island Group, Sample ID, Date (DD-MM-YYYY) of tissue biopsy, Lat/Lon, Sex (F = Female, M = Male, U = Unknown), Age class (A = Adult, J = Juvenile, U = Unknown), Disc Width, Catalog ID, and Catalog Name. The “n/a” indicates data was not obtained

Island Group	Sample ID	Date	Lat (°N)	Lon (°W)	Sex	Age Class	Disc Width (m)	Catalog ID	Catalog Name
Hawaiʻi Island	K26	03-03-2012	19.7347	-156.0567	F	A	3.39	247	Lefty MP184
Hawaiʻi Island	K27	03-03-2012	19.7347	-156.0567	F	J	2.5	705	Independence Ray MP84
Hawaiʻi Island	K28	09-05-2012	19.5585	-155.9669	F	J	2.89	706	Margo MP114
Hawaiʻi Island	K29	09-05-2012	19.5585	-155.9669	M	J	2.58	684	Eli Ray MP179
Hawaiʻi Island	K34	23-06-2015	19.5585	-155.9669	U	U	n/a	n/a	n/a
Hawaiʻi Island	K35	23-06-2015	19.5585	-155.9669	U	U	n/a	n/a	n/a
Hawaiʻi Island	K36	23-06-2015	19.5585	-155.9669	U	U	n/a	n/a	n/a
Hawaiʻi Island	K33	25-06-2015	19.5585	-155.9669	U	U	n/a	n/a	n/a
Hawaiʻi Island	K30	26-06-2015	19.5585	-155.9669	F	J	n/a	728	Amanda Ray
Hawaiʻi Island	K32	26-06-2015	19.5585	-155.9669	F	A	n/a	703	Vicky Ray
Hawaiʻi Island	K37	26-06-2015	19.5585	-155.9669	U	U	n/a	n/a	n/a
Hawaiʻi Island	K38	26-06-2015	19.5585	-155.9669	U	U	n/a	n/a	n/a
Hawaiʻi Island	K39	26-06-2015	19.5585	-155.9669	U	U	n/a	n/a	n/a
Hawaiʻi Island	K41	26-06-2015	19.5585	-155.9669	U	U	n/a	n/a	n/a
Hawaiʻi Island	K46	26-06-2015	19.5585	-155.9669	U	U	n/a	n/a	n/a
Hawaiʻi Island	K40	24-10-2015	19.5585	-155.9669	F	J	n/a	709	Lee Ray MP214
Hawaiʻi Island	K42	25-10-2015	19.5585	-155.9669	F	J	n/a	710	Winona MPO215
Hawaiʻi Island	K45	25-10-2015	19.5585	-155.9669	F	J	n/a	712	Akari MP218
Maui Nui	M24	30-01-2009	20.7913	-156.5880	F	A	3.37	231	PSI
Maui Nui	M25	02-04-2009	20.7913	-156.5880	M	A	n/a	59	Peace Right
Maui Nui	M02	25-11-2010	20.7913	-156.5880	F	A	3.37	102	Breakout
Maui Nui	M03	25-11-2010	20.7913	-156.5880	F	A	3.44	176	Bullseye
Maui Nui	M04	26-12-2010	20.7913	-156.5880	M	A	n/a	119	Boomerang
Maui Nui	M05	26-12-2010	20.7913	-156.5880	M	A	n/a	313	Solar
Maui Nui	M06	26-12-2010	20.7913	-156.5880	M	A	2.9	177	Pelvic Tats
Maui Nui	M07	26-12-2010	20.7913	-156.5880	M	A	n/a	82	Left Gifted
Maui Nui	M09	12-09-2012	20.7913	-156.5880	M	A	2.97	33	Sai
Maui Nui	M11	24-09-2012	20.7913	-156.5880	M	A	2.88	22	Salt Shaker
Maui Nui	M12	17-10-2012	20.7913	-156.5880	F	J	3.23	126	Cat Paw
Maui Nui	M14	07-11-2012	20.7913	-156.5880	M	A	2.94	13	Cluster
Maui Nui	M15	07-11-2012	20.7913	-156.5880	M	A	2.97	181	Coconut Split
Maui Nui	M16	24-02-2015	20.7913	-156.5880	F	J	2.83	150	Parentheses Right
Maui Nui	M17	17-05-2015	20.7913	-156.5880	F	A	3.41	16	Bee Hive
Maui Nui	M18	07-06-2015	20.7913	-156.5880	F	A	3.42	255	Cat Print
Maui Nui	M20	21-07-2015	20.7913	-156.5880	F	A	3.44	288	Staredown Right
Maui Nui	M21	21-07-2015	20.7913	-156.5880	M	A	2.96	191	Onion Dive
Maui Nui	M22	22-07-2015	20.7913	-156.5880	F	J	2.56	438	String Circle
Maui Nui	M23	19-10-2015	20.7913	-156.5880	F	J	3.13	154	Blowing Right

Surveys on Maui took place during the daytime, and primarily focused on a known manta ray cleaning station located at the south end of the Olowalu Reef (20.7913°N, -156.5880°W) and within a kilometer of the West Maui shoreline [9] (Table 7; Fig. 1). Hawaiʻi Island daytime surveys targeted manta rays opportunistically visiting cleaning stations or surface feeding in current lines within a kilometer off the West Hawaiʻi Island shoreline (Table 7; Fig. 1). Nighttime surveys were conducted at two popular commercial manta ray snorkel and dive locations at Keahou Bay (19.5585°N, -155.9669°W) and Makako Bay (19.7347°N, -156.0567°W) where manta rays feed on zooplankton attracted to underwater lights [14].

### Genetics benchwork

Whole genomic DNA was extracted from each sample using an Omega E-Z 96 Tissue DNA Kit (Omega), following the manufacturer’s protocol. DNA extracts were quantified using the AccuBlue High-Sensitivity dsDNA kit (Biotium, USA) on a Spectramax M3 fluorescent plate reader (Molecular Devices, USA) and visualized using gel electrophoresis. Samples were normalized (to 40ul) and 1-3ug of DNA per sample were digested overnight with DpnII restriction enzyme (NEB). We used the ezRAD approach [121] to construct restriction-associated digest (RAD) reduced representation libraries with DpnII (GATC cut site) following the ezRAD protocol [122] modified to a with-bead protocol (Additional file 3) using the Kapa HT TruSeq library preparation Kit (Roche Sequencing). In summary, fragmented DNA is end-repaired, 3’ ends adenylated, and ligated with Illumina TruSeq HT dual-indexed adapter sequences (IDT). DNA fragments from 300 to 425 bp (target insert sizes 200-300 bp) were isolated using a PippenPrep automated electrophoresis system (Sage Science). Adapter-ligated, size-selected fragments were then amplified using PCR (see Additional File 3 for conditions). Following each step, samples were cleaned using AMPureXP paramagnetic beads (Beckman-Coulter), which were left in the cleaned samples and reactivated by adding 2.5 M NaCl 20% PEG (Polyethylene glycol) to the solution at various steps [123]. DNA concentration was quantified following each step using Accublue Quantitation (Biotium). A Bioanalyzer (Applied Biosystems) was used to check size-distribution and quality of final amplified libraries. Individually barcoded libraries were normalized in equimolar concentrations (150ng) and combined in equal proportions into a single library per island. The two pooled libraries were cleaned with a final 1:1 bead cleanup and sequenced on one lane of an Illumina HiSeq 3000 (PE 150) at the UCLA Technology Center for Genomics and Bioinformatics. Raw sequenced reads were demultiplexed by index and barcode by the sequencing facility, and only samples with matched index pairs were retained, thereby eliminating index mis-assignment. Sequencing produced 268.4 million raw 150 bp sequences across 46 libraries (Hawaiʻi Island = 21; Maui Nui = 25) of *Mobula alfredi*. We recovered 9,872 to 16,825,862 reads (5.8 ± 4.2 million; mean ± sd) per individual library (Table S1).

### Mitogenome assembly and haplotyping

Due to the high prevalence of the GATC cut site in mitochondrial genomes combined with stochastic fragmentation, whole mitogenomes can be assembled from ezRAD libraries [40, 121, 124, 125]. We assembled the complete *M. alfredi* mitogenome (including the control region) from individual M03 from Maui (OP562409.1, described in [40]), which we used as the reference for aligning and haplotyping. Raw reads were subject to QA/QC, adapter trimming, and mate-pairing validation as described below for nuclear SNPs. Cleaned paired reads for 38 individual *M. alfredi* (Hawaiʻi Island = 18, Maui Nui = 20) were aligned to this reference mitogenome using BWA v.0.7.17 [126], Samtools v.1.6 [127], and Bamtools v.2.5.2 [128]. Freebayes v.1.2.0 [129] was used to call variant sites on all haplotype alleles (including reporting on all monomorphic sites) with a minimum of 4x coverage to call. These settings included: ploidy = 1, polymorphic site must be variable in at least 2 reads, minimum quality score 20, minimum mapping quality 15, minimum coverage of 4x. After filtering for low depth individuals (missing calls > 60% of sites), 34 individuals remained in the dataset. On average, those individuals had 82.6x coverage across 77.1% of the mitogenome, mean read depth from 16x to 366x, and high-quality base calls (≥ 4x) between 62.4 and 99.4% of the mitogenome (Additional file 4). Before filtering, we identified 62 potential variable sites (at ≥ 4x) across the mitogenomes: 11 of which were shared among two or more individuals, while the majority (51) were present in a single individual (singletons). Many of the singleton sites had low read coverage and/or multiple alleles present per individual (i.e., appear as heterozygotes), which strongly indicate sequencing or mapping errors when haploids. We evaluated the relationship between the number of variable sites and the minimum read depth to determine a variant site filtering threshold (Fig. S1). We observed that the number of shared variable sites was relatively constant across read depths while the number of singleton sites decreased with increasing coverage and plateaus at a minimum average read depth of ~ 10x. Therefore, in selecting mitogenome variant sites for analysis we applied a minimum average coverage threshold of 10x, which resulted in 12 potential variant sites. We applied one additional filter using mapping discrepancies between the reference and the alternate alleles, which filtered three singletons with high coverage of both reference and alternate alleles in a single individual (i.e., appeared as heterozygotes) and are likely due to sequencing/mapping errors in haploids. The application of these two filters resulted in a final set of nine variant sites across the mitogenome, including eight shared mutations (parsimony informative) and one singleton site. We then used a less stringent coverage threshold of 4x to call alleles at those nine variant sites. We then extracted mitogenome haplotypes into fasta files for 34 individuals using VCFTools v.0.1.12a [130] and masked sites with less than 4x coverage with N’s.

#### Mitogenome data analysis

We calculated molecular diversity indices using DnaSP v.6.12.01 [131]. We imputed missing data in genodive v.2.0b27 [132], using population specific allele frequencies. There was a total of 41 missing allele calls (13.4%) out of 306 alleles (34 individuals x 9 variable sites). Imputed missing data were not used for any other tests or inference outside of estimating diversity indices in DnaSP. Neutrality tests, Tajima’s *D* [133] and Fu’s *F*_*s*_ [134] were performed using arlequin v.3.5.2.2. [135] to infer demographic history. Analyses of molecular variance (AMOVA) were conducted in arlequin, to test for genetic structure among individuals from different islands and estimate the degree of genetic differentiation (*Φ*_*ST*_) among islands, using a Tamura-Nei model of nucleotide evolution (selected by jModelTest 2 [136]). Using 10,000 permutations, we calculated pairwise *Φ*_*ST*_ for the whole mitogenome and separately for five individual genes with variable sites including four coding regions (16S, NADH4, NADH5, cytochrome *b*) and the control region. A haplotype network was constructed for the mitogenome with network v.4.6.1.1 [137] using a median-joining algorithm [138] and default settings.

### Nuclear SNP discovery and genotyping

Our nuclear SNP discovery and genotyping workflow included five major steps: (1) quality filtering (adapter cleaning and mate-pair validation), (2) de-novo pseudo-reference generation, (3) alignment to pseudo-reference RAD contigs, (4) variant calling, and (5) SNP quality control filtering. For quality filtering, raw reads were assessed using FastQC [139] and reads with sequencing adapters present were filtered out using Cutadapt v1.11 [140] with maximum error rate (10%), minimum overlap 12 bases, and search algorithm (-b; found anywhere in the sequence). Orphaned reads were removed leaving only mate-paired reads. A total of 134,119,542 mate-paired reads remained after adapter cleaning and quality filtering (Table S1).

#### De-novo pseudo-reference generation

A pseudo-reference of ezRAD loci for *Mobula alfredi* was generated *de novo* using Seanome [141]. In short, we input sequences from 4 individual reef manta rays (two from each island group) with the highest sequence read count following cleaning (each with 4–6 million reads). Properly mated paired reads were merged using Pear v.0.9.10 [142] using default settings and minimum assembly length of 100 bases. Then commonly shared regions (CSRs) were found using minimum length of 100 bases for a shared region (contig), and minimum similarity of 95% to be clustered together. A total of 22,098,270 merged reads from four individuals were assembled into 872,500 commonly shared regions between 100 and 650 bases (mean = 202 bases; 99% between 100 and 300 bases) with an average of 25x coverage. We then used Usearch v8.1 [143] to remove duplicate and reverse complement contigs and cluster unique sequences with 95% similarity (centroids) into 734,031 clusters. We then used mothur v.1.4.3 [144] to exclude contigs: with homopolymers ≥ 8 bases, ambiguities (i.e., no Ns), lengths < 200 bases, and that do not start and end with restriction enzyme cut site (GATC). These filtering steps produced a final output of 359,756 reference contigs (RAD loci) with an average length of 220 bases (range 200–529) and a total length of 79,380,335 bases. We then used local BLASTn to query pseudo-reference contigs (359,756) to the *M. alfredi* mitogenome (GenBank Accession: OP562409). Five contigs matched well to the mitogenome reference (98–100% identity), all with Scores > 400, very low E-values (1e-150) and overlapping lengths between 271 and 337 bases. We checked if any of these five contigs matched with the final post-filtering SNP set contigs (2048 SNPs on 459 contigs) and none were present. Therefore, this confirms the final SNP set contains only nuclear loci, with no hits to the mitogenome reference > 100 bases and an e-score > 1e-20.

#### Alignment, variant calling, and SNP filtering

The dDocent pipeline [145] was used to map reads with BWA and call variants with FreeBayes. We indexed our reference contigs using Samtools, BWA, and Picard tools v.1.102 [146]. Fastq sequences were adapter-cleaned, mate-paired, and all filtered to minimum length of 150 bases. Eight libraries with too few reads (< 150,000 cleaned-mated reads) were excluded from alignments and all downstream analyses (Table S1). Sequences from the 38 remaining individuals were aligned to our pseudo-reference with BWA (BWA-mem, paired sequences) with settings based on dDocent recommendations. Using Samtools we extracted only properly paired mappings and excluded unmapped reads. We performed variant calling with FreeBayes using the following settings: minimum mapping quality (5), minimum quality score (15), read-max-mismatch fraction (0.2), mismatch-base-quality threshold (10), read indel limit (5), min-alt total (10), read mismatch limit (20), and max 4 alleles.

SNPs in nuclear RAD loci were extensively filtered before analysis using a workflow (see Additional file 3 for detailed SNP filtering workflow) based on the dDocent protocol [145] and following recommendations from O’Leary et al. [147] and Portnoy et al. [44]. In summary, the initial dataset of 49,028 SNPs output from FreeBayes was first filtered to remove all genotypes with < 5 reads per individual, quality scores < 25, and loci called in < 50% of individuals. SNPs were then filtered to meet the following criteria: called in 85% of all individuals (i.e., < 15% missing data per SNP); minor allele count > 2, minor allele frequency > 5% across all individuals, and conforming to expectations of Hardy-Weinberg equilibrium (HWE; *P* < 0.01 in both populations). This included the removal of SNPs with: low quality-to-depth ratios (< 0.25), discrepancies between mapping qualities and properly paired status of reference and alternate alleles, mean read depth < 20 and > 120x; and the removal of RAD loci (contigs) with excess SNPs (> 24) and those identified as possible paralogs. The final nuclear SNP set contained 2048 SNPs across 459 RAD loci (mean 4.5 SNPs per RAD locus) with 38 individuals (Table 7) containing high quality genotypes called in > 95% of all sites (i.e., < 5% missing genotypes per individual). Raw variants were filtered sequentially using VCFtools, VcfLib v.1.0.3 [129, 148], Rad Haplotyper v.1.1.9 [149] and dDocent bash scripts from Puritz et al. [145] detailed steps in Additional file 3. VCF and other file format conversions were executed using PGDSpider v.2.0.8.3 [150]. Relatedness of individuals was assessed in VCFtools, using the statistic of Yang et al. [151]. The average relatedness among individuals was − 0.02 and no pairwise comparison had a relatedness greater than 0.15. We conclude that the final genotype set has no closely related individuals.

#### Outlier loci detection and annotation

We assessed population structure using three nuclear SNP datasets: all loci (2048), neutral loci (2038), and outlier loci (10), which allowed us to make inferences regarding selection. To identify neutral and outlier loci we used the R package OutFLANK [152, 153], which estimates a null distribution of *F*_ST_ for loci unlikely to be under strong positive selection. We ran OutFLANK implementing a left and right trim factor of 0.05, a minimum heterozygosity of < 10%, and a false discovery rate of 5% (q = 0.05). The contigs (RAD loci) containing outlier SNPs identified using OutFLANK were used as queries (e-value < 10^− 6^) against the nr database at the National Center for Biotechnology Information (NCBI), using Blast2GO Pro [154] to find homologous sequences, map and annotate Gene Ontology (GO) terms (e-value < 10^− 6^, annotation cut-off > 55 and a GO weight > 5).

### Characterizing island populations

Genodive was used to generate genetic diversity indices for all three datasets, as well as to test for population structure. Genetic structure among sample locations was evaluated with an analysis of molecular variance (AMOVA) in arlequin. Deviations from null distributions were tested with non-parametric permutation procedures (N = 9999). Pairwise *F*_ST_ statistics were generated to assess genetic structure between locations. False discovery rates were controlled for and maintained at α = 0.05 among all pairwise tests [155, 156]. Genetic partitioning was assessed for all datasets using structure v.2.3.2 [157], a Bayesian method that estimates ancestry and categorizes individuals into discrete populations. Sampling location was provided for each individual and set as priors; the admixture model and allele frequency correlated model were implemented in structure to assess ancestry. The simulation was run for 1 million generations with the first 100,000 discarded as burn-in. Five replicates of each simulation from K = 1 to 5 genetic clusters were run. We determined the most likely number of genetic clusters (K) indicated from the Evanno method [158] and selecting the clusters inferred from delta K vs. K in structure harvester v.0.6.93 [159]. Structure results were analyzed and visualized using the on-line tool CLUMPAK [160], which integrates the program clumpp v.1.1.2 [161] and minimizes the variance across all iterations. We also tested population structuring for the neutral and outlier loci datasets using a discriminant analysis of principal components (DAPC) in Adegenet v4.0.2 for R [162]. We imposed the number of clusters (K) of two and ran analyses without any priors for both neutral and outlier loci. Each DAPC was performed using one discriminant function, which is the maximum when K = 2, and the optimal number of principal components (12 for neutral loci and 1 for outliers) was chosen using the a-spline optimization procedure [162]. Contemporary effective population size estimates for each island were calculated for each of the datasets using the molecular co-ancestry method of [163] as implemented in NeEstimator v.2.1 [164]. Here, we use the point random mating Linkage Disequilibrium (LD) method and report estimates for critical values (the criterion for excluding rare alleles = 0.05). We applied a physical linkage correction factor following Venables et al. [34] and report both the raw and adjusted estimates of *N*_*e*_, and the parametric 95% confidence levels.

#### Direction and magnitude of migration among islands

To examine the direction and magnitude of migration between Maui Nui and Hawaiʻi Island, migration rates were calculated using MIGRATE-N v.4.4.3 [165, 166] separately on both the neutral nuclear loci (2038 SNPs) and mitochondrial haplotypes. Several test runs were conducted to determine the appropriate prior values for the parameters *θ* (four times effective population size multiplied by mutation rate per site per generation, *4Neµ*) and *M* (immigration rate divided by the mutation rate, m/µ). In the final analyses the mean prior values for *θ* and for *M* were set in both directions (i.e., between islands). After checking for data convergence, the mode and 95% percentiles of *θ* and *M* were used to calculate the effective number of migrants per generation (*M*_*e*_) between populations to determine the direction and magnitude of migration. A relative migration network was constructed in *R* using the *divMigrate* function in the *diveRsity* package [167, 168] and implemented using Jostʻs *D* [169] statistical method, based on 1000 bootstrap replicates. We took the inverse migration rate (1/*M*_*e*_) to calculate the number of generations needed to achieve 1 effective migrant between populations, then multiplied by the estimated generation time for *M. alfredi* (29 years; [10, 25, 28]) to provide an approximate number of years every 1 individual effectively migrates between populations.

## Electronic supplementary material

Below is the link to the electronic supplementary material.

**Additional file 1** Supplementary figures S1-S4.

**Additional file 2** Supplementary tables S1-S6.

**Additional file 3** (1) Step-by-step protocol for library prep benchwork and (2) Detailed steps of SNP filtering workflow

**Additional file 4** Mitogenome Assembly & Alignment Summary Tables

## Data Availability

The datasets supporting the conclusions of this article are available on Zenodo 10.5281/zenodo.7312296 or are included within the article (Additional files). Mitogenome reference was deposited in GenBank (Accession: OP562409; [40]). Raw sequences were deposited in NCBI’s Sequence Read Archive: https://www.ncbi.nlm.nih.gov/sra/PRJNA885488; SRA Accessions: SRR21884470-SRR21884507; BioProject https://www.ncbi.nlm.nih.gov/bioproject/885488.
