# Supplementary material for: Genomic evidence indicates small island-resident populations and sex-biased behaviors of Hawaiian reef Manta Rays
Source: BMC Ecol Evol. 2023 Jul 8;23:31. doi: 10.1186/s12862-023-02130-0 (PMC10329317; doi:10.1186/s12862-023-02130-0)
Supplement: Supplementary file 1 — Additional file 1 Supplementary figures S1-S4. [file 12862_2023_2130_MOESM1_ESM.docx]

**Supplementary Information**

for

“Genomic evidence indicates small island-resident populations and sex-biased behaviors of Hawaiian reef manta rays”

Jonathan L. Whitney^1,2*^, Richard R. Coleman^3^, and Mark H. Deakos^4^

^1^ National Oceanic and Atmospheric Administration, Pacific Islands Fisheries Science Center, Honolulu, Hawaiʻi

^2^ Department of Oceanography, University of Hawaiʻi at Manoa, Honolulu, Hawaiʻi

^3^Rosenstiel School of Marine and Atmospheric Science (RSMAS), University of Miami, Miami, Florida

^4^ Hawaii Association for Marine Education and Research, Lahaina, Maui

^*^Corresponding author. Email: [Jonathan.Whitney@noaa.gov](mailto:Jonathan.Whitney@noaa.gov); Jw2@hawaii.edu


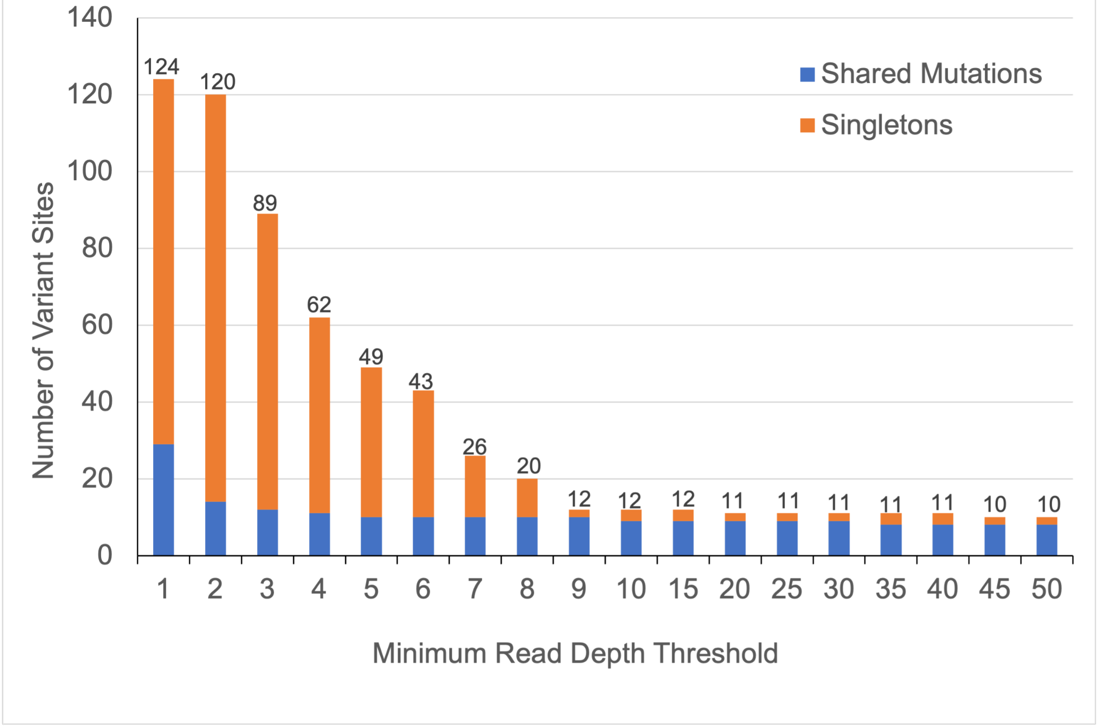


**Figure S1**. Number of variable sites across 34 *Mobula alfredi* mitogenomes with respect to minimum mean read depth threshold, grouped by singletons (orange) and shared mutations (blue).


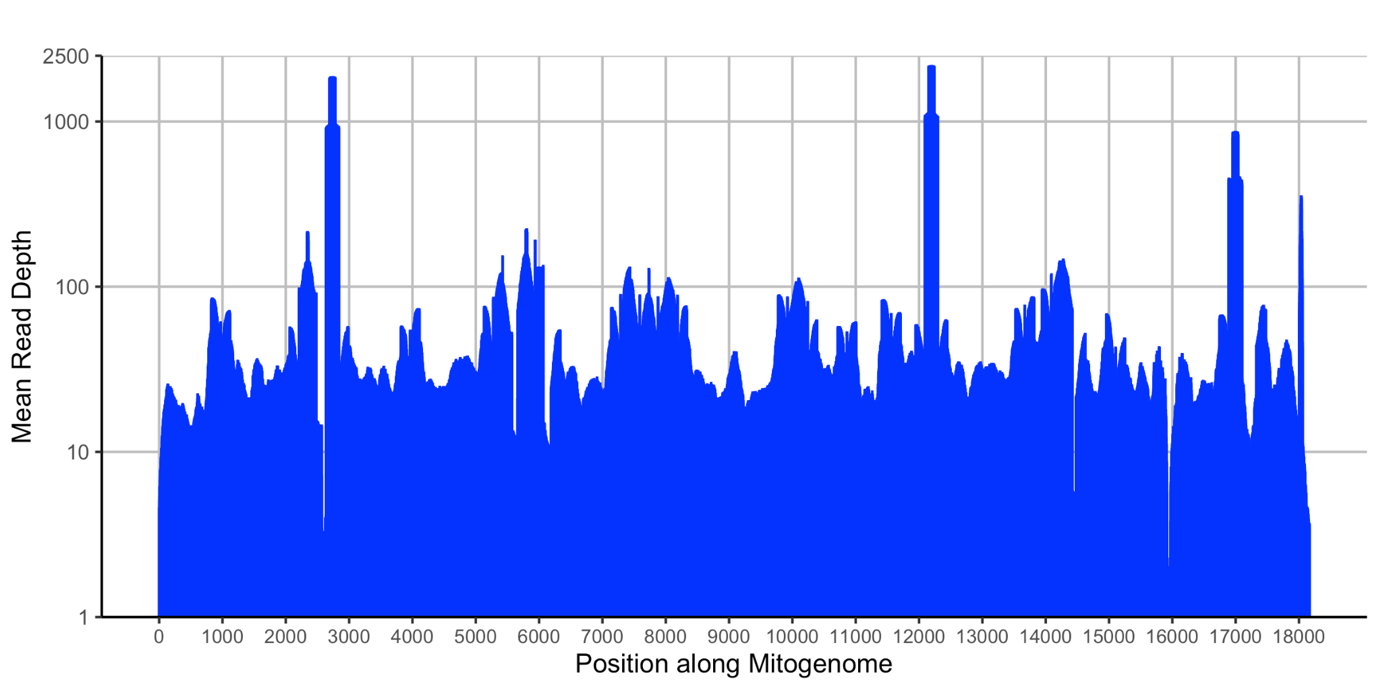


**Figure S2**. Mean read depth along mitogenome across 34 *Mobula alfredi* individuals mapped to assembled mitogenome (Genbank: OP562409).


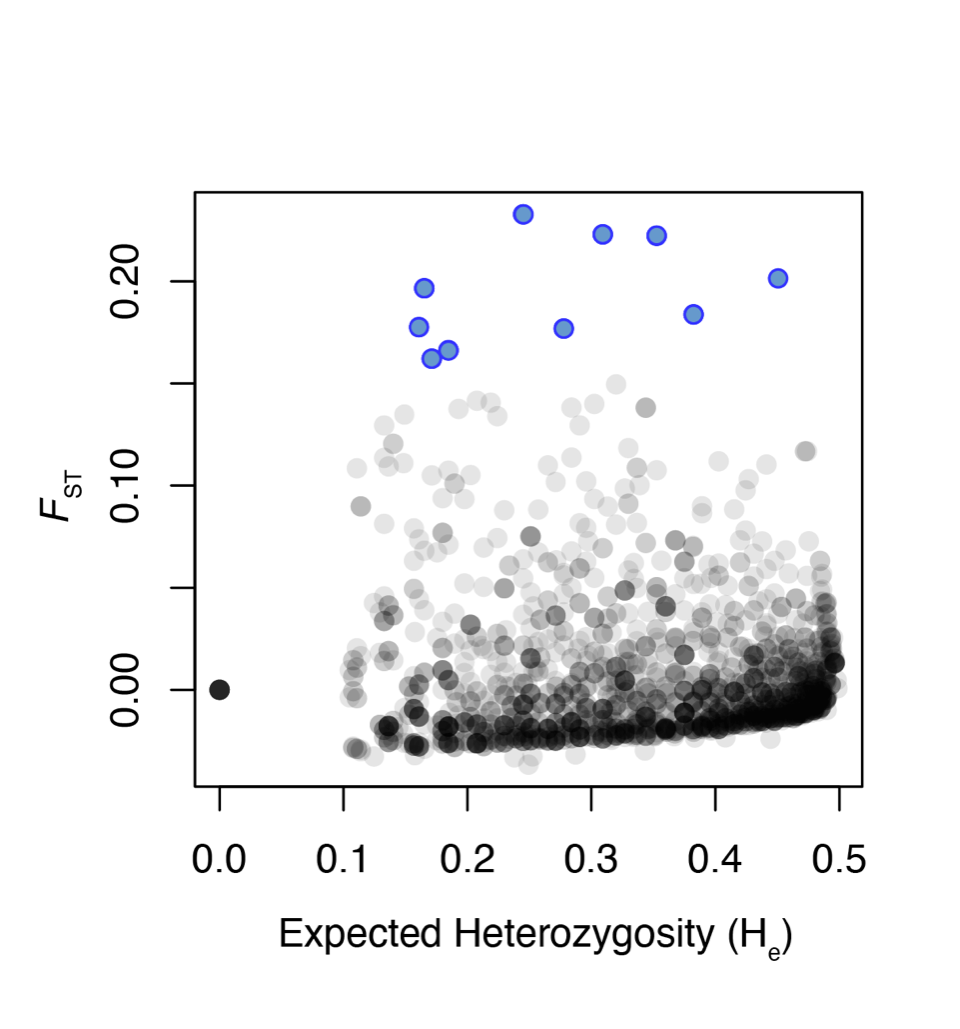


**Figure S3.** Genetic differentiation versus expected heterozygosity of 2048 nuclear SNPs genotyped. In the scatterplot, black and grey dots indicate neutral loci (n=2038), and blue dots indicate outlier loci (n=10).

A)


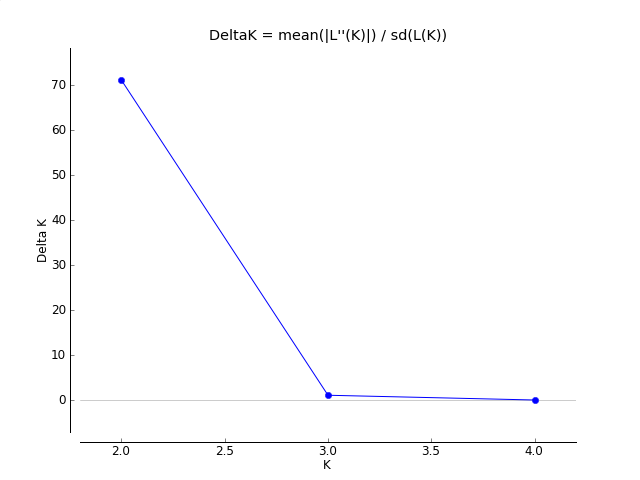


B)


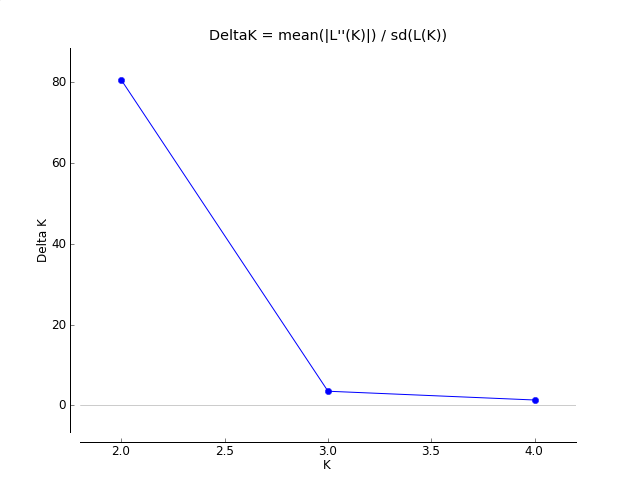


C)


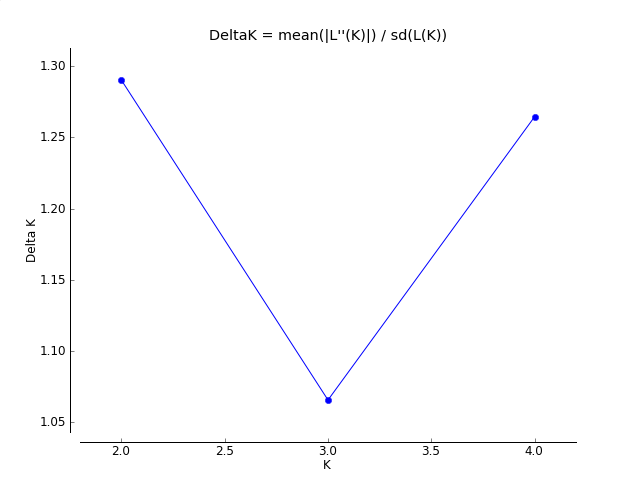


**Figure S4.** DeltaK graph generated from STRUCTURE HARVESTER (Earl & von Holdt 2012). A) all loci, 2048 snps, B) neutral loci, 2038 snps, C) outlier loci, 10 snps.
