# Supplementary material for: Genomic evidence indicates small island-resident populations and sex-biased behaviors of Hawaiian reef Manta Rays
Source: BMC Ecol Evol. 2023 Jul 8;23:31. doi: 10.1186/s12862-023-02130-0 (PMC10329317; doi:10.1186/s12862-023-02130-0)
Supplement: Supplementary file 3 — Additional file 3 (1) Step-by-step protocol for library prep benchwork and (2) Detailed steps of SNP filtering workflow [file 12862_2023_2130_MOESM3_ESM.docx]

**Additional file**

for

“Genomic evidence indicates small island-resident populations and sex-biased behaviors of Hawaiian reef manta rays”

Jonathan L. Whitney^1,2*^, Richard R. Coleman^3^, and Mark H. Deakos^4^

^1^ National Oceanic and Atmospheric Administration, Pacific Islands Fisheries Science Center, Honolulu, Hawaiʻi, USA

^2^ Department of Oceanography, University of Hawaiʻi at Manoa, Honolulu, Hawaiʻi, USA

^3^Rosenstiel School of Marine and Atmospheric Science (RSMAS), University of Miami, Miami, Florida, USA

^4^ Hawaii Association for Marine Education and Research, Lahaina, Maui, USA

With-Bead Library prep Protocol using Kapa HT TruSeq Kit (1/2 volumes)

(modified from Faircloth et al. 2014 – Illumina Prep v2.1 (BadDNA.org)

1. Concentrate genomic DNA (up to 3 ug) to 40 uL in centrivac (add H2O if <40 uL)

**Restriction Enzyme Digest**

1. Assemble RE digest master mix:

| 1X DpnII Buffer | 5 μL |
| --- | --- |
| DpnII (20 units) | 2 μL |
| Purified gDNA | 43 μL |
| **Total** | **50 μL** |

1. Add **7 uL digest master mix** to 43 uL gDNA and mix by pipetting (50 uL total volume).
2. Incubate in Thermocycler overnight (16hr) at 37 °C, then inactivate at 65 °C for 20 minutes, hold at 4 °C ∞
3. Run 1 uL of sample on gel (2%, 100 V for 55 min) to check digestion worked.

-------------------------------------------------SAFE STOPPING POINT-----------------------------------------------------------

**Post-digestion Cleanup**

1. Add **150 uL AmpureXP Beads** (3x Volume) to 50 uL digested DNA and mix by vortezing (200 uL total volume).
2. Conduct Ampure Cleaning Protocol.
3. Resuspend beads with **42.5 uL ddH2O** and **proceed immediately** to End Repair.

**End Repair**

1. Assemble the end-repair master mix:

| 10X End Repair Buffer | 5 μL |
| --- | --- |
| End Repair Enzyme Mix | 2.5 μL |
| **Total** | **7.5 μL** |

1. Add **7.5 uL** **End-repair master mix** to 42.5 uL bead/water/DNA solution and mix gently by pipetting (50 uL total volume).
2. Incubate in Thermocycler for 30 min @ 20 °C; hold at 10 °C or 4 °C for longer.

-------------------------------------------------SAFE STOPPING POINT-----------------------------------------------------------

**Post End Repair Cleanup**

1. Add **85 uL PEG (**1.7X) solution to 50 uL end-repair product and mix by pipetting or gently vortexing (135 uL total volume).

(Note: Bird uses 1.5X volume)

(PEG solution = 20% Polyethylene glycol (MW 8000), 2.5 M NaCl).

1. Conduct Ampure Cleaning Protocol and **proceed immediately** to A-tailing reaction.

**A-tailing Reaction**

1. Assemble the A-tailing reaction mix:

| 10X A-tailing Buffer | 2.5 μL |
| --- | --- |
| A-tailing Enzyme | 1.5 μL |
| ddH2O | 21 μL |
| **Total** | **25 μL** |

1. Add **25 uL A-tailing** **master mix** to dried beads and mix gently by pipetting.
2. Incubate in Thermocycler for 30 min @ 30 °C.
3. Add **45 uL PEG** solution (1.8X) to 25 uL reaction with beads and mix by pipetting or gently vortexing. (80 uL total volume).
4. Conduct Ampure Cleaning Protocol and **proceed immediately** to adapter ligation.

**Adapter-Ligation**

1. Assemble the adapter ligation master mix:

| 5X Ligation Buffer | 5 μL |
| --- | --- |
| DNA Ligase | 2.5 μL |
| ddH2O | 15 μL |
| **Total** | **22.5 μL** |

1. Add **22.5 μL ligation master mix** to dried beads and mix gently by pipetting.
2. Add **2.5 uL sample-specific Adapter* Mix** (30 uM) to the 22.5 uL bead/ligation mix solution and mix gently by pipetting.
3. Incubate for 15 min @ 20 °C, and proceed immediately to cleanup.

*Adapter Sequences

Illumina TruSeq HT dual-indexed adapters

GATCGGAAGAGCACACGTCTGAACTCCAGTCACNNNNNNNNATCTCGTATGCCGTCTTCTGCTTG

GATCGGAAGAGCGTCGTGTAGGGAAAGAGTGTNNNNNNNNGTGTAGATCTCGGTGGTCGCCGTATCATT

**Post-Ligation Cleanup**

1. Add **25 uL PEG** (1.0X) mixture to 25 uL ligation product and mix gently by pipetting or vortexing (50 μL total volume). We’re using less PEG solution at this step to help us remove very short fragments.
2. Conduct Ampure Cleaning Protocol.
3. Resuspend dried beads in **35 uL ddH20** and mix by pipetting. We will clean these again.
4. Add **35 uL PEG** solution (1.0X) mixture to reaction with beads. Mix by pipetting or gently vortexing. We’re using less PEG solution at this step to help us remove very short fragments.
5. Conduct Ampure Cleaning Protocol.
6. After drying, resuspend beads in **35 uL elution buffer (10 mM Tris-Acetate, pH 8)**, and incubate at room temp for 2-5 mins to release DNA from beads.
7. Capture beads by placing on magnet at room temp for 3-5 minutes or until solution is clear.
8. Recover the DNA in **35 μL** of supernatant and transfer to the tube/well and store at -20 °C.
9. Perform second elution on remaining beads in 35 uL elution buffer and store @ -20 **°**C for troubleshooting.

-------------------------------------------------SAFE STOPPING POINT-----------------------------------------------------------

**Size-Selection**

1. Use Pippen Prep to conduct size selection (30 μL input results in 40+ μL of size-selected library).

**Amplification**

1. Quantify 2 μL using Accublue (or QuBit)
2. Centrivac samples from 40 uL to 20 uL – and use half (10ul) for amplification.
3. Assemble the PCR master mix:

| 2X Kapa HiFi HotStart Master Mix | 12.5 μL |
| --- | --- |
| Primer Mix (2.5 uM each F + R primers) | 2.5 μL |
| **Total** | **15 μL** |

1. Add **15 uL PCR master mix** to **10 uL Adapter-ligated, size-selected DNA** (25 uL total volume)
2. Cycle using the following. You may need to adjust or optimize cycle number (6-8)

• 98 C for 45 sec

• 6-12 cycles of: 98 C for 15 sec, 60 C for 30 sec, 72 C for 60 sec

• 72 C for 5 minutes

**Final Cleaning of PCR Product**

1. Add **45 uL AMPure XP beads** (1.8X) to the 25 uL PCR product. Mix by pipetting or vortexing.
2. Conduct Ampure Cleaning Protocol.
3. Resuspend dried beads in **24 uL elution buffer (10 mM Tris-Acetate, pH 8)**, incubate at room temp for 2-5 mins to elute DNA.
4. Capture beads by placing on magnet at room temp for 3-5 minutes or until solution is clear.
5. Pull off **24 μL** of supernatant DNA and transfer to a new tube/well. This is the “pure gold” adapter-ligated, size-selected, amplified and cleaned DNA library to go to qPCR/sequencing.
6. Perform second elution on remaining beads in 30 uL elution buffer and store @ -20 **°**C for troubleshooting.

**Ampure Cleaning Protocol**

1. Incubate the mixture for 5 mins.
2. Move tubes to magnet & let sit for 3 mins (until solution is clear).
3. Aspirate liquid from tubes and discard (save supernatant for troubleshooting).
4. Add 200 uL 80% EtOH to each tube in the stand and incubate 30 seconds
5. Aspirate liquid from tubes and discard.
6. Add 200 uL 80% EtOH to each tube in the stand and incubate 30 seconds
7. Aspirate EtOH from tubes and discard.
8. Allow beads to dry for ~ 5 minutes or until there is no longer a smell of EtOH.

SNP Filtering Steps

Raw variant calls from Freebayes were subject to several filtering steps. The SNP filtering workflow we implemented is based on the dDocent protocol (Puritz et al. 2014a) with further recommendations from O’Leary et al. (2018) and Portnoy et al. (2015). The initial raw SNP VCF dataset output by FreeBayes contained 49,028 SNPs in 40 individuals (n=20 in each population). Raw variants were filtered sequentially using VCFtools (Danecek et al. 2011), vcffilter in VcfLib (Garrison 2012, https://github.com/vcflib/vcflib), Rad Haplotyper (Willis et al. 2017) and dDocent bash scripts from Jon Puritz and Chris Hollenbeck using the following steps:

- 1. Removed loci with minor allele count less than 2, a PHRED quality score less than 25, and a call rate of less than 50%.
  2. Genotypes with less than 5 reads were changed to missing.
  3. Removed loci with genotype call rate <85% (i.e., removed loci with more than 15% missing data), and minor allele frequencies (MAF) < 5% across all populations, and a minimum mean depth of 20x.
  4. Removed loci with large discrepancies between mapping qualities of reference and alternate alleles. Specifically, loci were retained if the ratio of mapping qualities of alternate (MQM) and reference alleles (MQMR) were between 0.9 and 1.05.
  5. Removed loci with large discrepancies in the properly paired status of reference (PAIREDR) and alternative alleles (PAIRED). This is based on the proportion of alternate/reference alleles which are supported by properly paired read fragments. False variants tend to have properly paired reference reads but not properly paired alternate reads. Specifically, loci were removed if either proportion (PAIREDR, PAIRED) is less than 0.05 or if the ratio of proportions (PAIREDR/PAIRED) is greater than 1.75 or less than 0.25. (i.e., if proportion of properly paired reads for reference alleles (PAIREDR) is >3-times higher or lower than then proportion of properly paired reads for alternate alleles (PAIRED).
  6. Removed any locus with quality score (QUAL) below ¼ of the depth (DP) (i.e., ratio of QUAL/DP < 0.25).
  7. Removed loci with that have read depth greater than $d+2*\surd d$, whereby *d* is the average read depth. This has been shown to be effective in reducing paralogs and false heterozygotes from sequencing errors (Li 2014).
  8. Removed loci with very high coverage (mean read depth >120x), which are more likely to be paralogs or multicopy loci. This resulted in a set of SNPs with average read depths between 20x and 120x.
  9. Variant calls were decomposed into phased SNP and INDEL genotypes using (vcfallelicprimitives).
  10. Removed Indels to create a SNP only dataset.
  11. SNP loci not meeting expectations of Hardy-Weinberg equilibrium were removed (i.e., loci with P-value less than 0.01 in at least 50% of populations) using the script filter_hwe_by_pop.pl (<https://github.com/jpuritz/dDocent/raw/master/scripts/filter_hwe_by_pop.pl>)
  12. Using RAD Haplotyper to identify loci with excess SNPs (>24) and possible paralogs, which were then removed using the script (remove.bad.hap.loci.sh):

(<https://raw.githubusercontent.com/chollenbeck/rad_haplotyper/master/rad_haplotyper.pl>).

<https://github.com/jpuritz/dDocent/raw/master/scripts/remove.bad.hap.loci.sh>

- 1. Final filtration by max missing 15% and excluding individuals genotyped at less than 50% SNP loci. The final dataset contained 38 individuals (Kona 18, Maui 20) all with <5% missing data (i.e., high quality genotypes called in >95% of all 2048 SNPs).

**Supplemental References:**

Danecek P, Auton A, Abecasis G, *et al.* 2011. The variant call format and VCFtools. *Bioinformatics*, 27, 2156-2158.

Garrison E. 2016. Vcflib, a simple C++ library for parsing and manipulating VCF files.. <https://github.com/vcflib/vcflib>.

Li H. 2014. Towards better understanding of artifacts in variant calling from high-coverage samples. *Bioinformatics*, 30, 2843-2851.

O'Leary SJ, Puritz JB, Willis SC, Hollenbeck CM, Portnoy DS. 2018. These aren’t the loci you’re looking for: Principles of effective SNP filtering for molecular ecologists. *Molecular Ecology* 27:3193–3206.

Portnoy D, Puritz JB, Hollenbeck CM, Gelsleichter J, Chapman D, Gold JR. 2015. Selection and sex‐biased dispersal in a coastal shark: The influence of philopatry on adaptive variation. *Molecular Ecology* 24, 5877-5885.

Puritz JB, Hollenbeck CM, Gold JR. 2014. dDocent: a RADseq, variant-calling pipeline designed for population genomics of non-model organisms. *PeerJ*, 2, e431.

Puritz JB, Matz MV, Toonen RJ, Weber JN, Bolnick DI, Bird CE. 2014. Comment: Demystifying the RAD fad. *Molecular Ecology* 23: 5937–5942.

Willis SC, Hollenbeck CM, Puritz JB, Gold JR and Portnoy DS. 2017. Haplotyping RAD loci: an efficient method to filter paralogs and account for physical linkage. *Molecular Ecology Resources* 17: 955–965.
